# Fluorinated carbohydrate-based vaccines†

**DOI:** 10.1039/d6sc02383e

**Published:** 2026-05-21

**Authors:** Elena Chikunova, James Suri, Kathrin Siebold, Ryan Gilmour

**Affiliations:** a Institute for Organic Chemistry, University of Münster Corrensstraße 36 48149 Münster Germany ryan.gilmour@uni-muenster.de

## Abstract

The structural virtuosity of carbohydrates forms the basis of a molecular recognition language that is ubiquitous in immunomodulatory processes. This diversity generates the complexity required to accommodate the bandwidth of information generated by immune interactions: it logically follows that this capacity underpins the evolutionary success of oligosaccharide permutations in this endeavour. Placing glycan function on a structural level is therefore a core research endeavour in understanding the non-covalent interactions that elicit an immune response and, by extension, leveraging these data to identify and validate new vaccine leads. Innovations to expedite the construction of well-defined, immuno-relevant carbohydrates has revolutionized the field, and focused attention on further structural refinements that are new to biology. Echoing the success of site-selective fluorination in pharmaceutical design, the precision introduction of C(sp^3^)–F bonds to tailor the physicochemical properties of key targets is an exciting new frontier in targeted immunology. In this short Perspective, the key milestones in the design and validation of carbohydrate-based vaccine leads are discussed through the lense of fluorination.

## Abstract 2

The structural virtuosity of carbohydrates forms the basis of a molecular recognition language that is ubiquitous in immunomodulatory processes.

## Graphical abstract

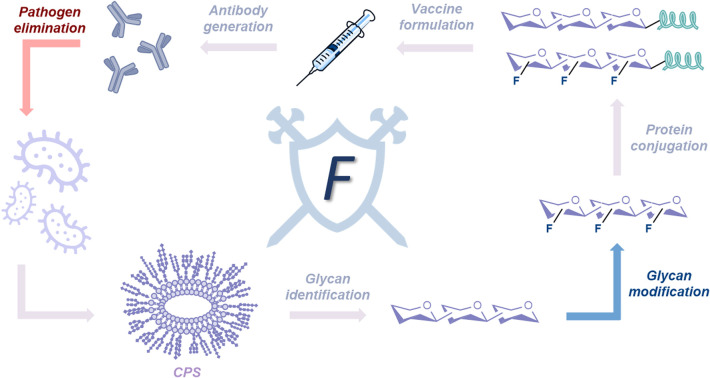

## 1. Introduction

**Fig. 1:**
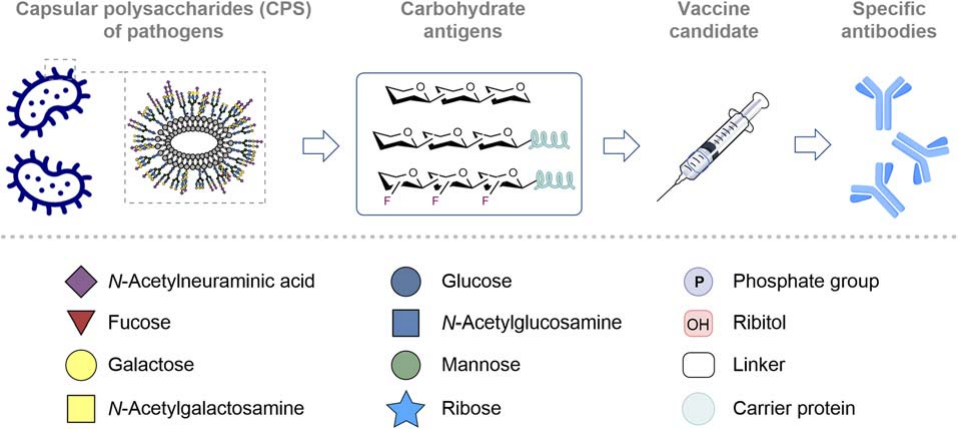
Top: General principles of carbohydrate-based vaccine design. Identification and isolation or synthesis of the target glycans from the target pathogen. Glycan conjugation to an immunogenic carrier protein. Creation of a vaccine formulation that may also include an adjuvant. Following administration, the recipient produces antibodies specific for the target pathogen; bottom: a key for symbol nomenclature used throughout this perspective.

Vaccination is a fundamental pillar of public health and the legacy of Salk's totemic introduction of a safe and effective polio vaccine in the mid-1950s.^1–3^ Its impact is reflected in the World Health Organization's (WHO) estimate that vaccination programmes currently prevent some 3.5–5 million deaths per year worldwide.^4^ This approach to disease prevention and management has played a significant role in reducing the global under-five mortality rate,^5–7^ thereby lessening the risk posed by numerous fatal infections. Success has been grounded in centuries of observation, experimentation, failure, and persistence, resulting in the aforementioned (r)evolution.^8–11^ In response to these efforts, pathogens also continually evolve, developing resistance to vaccines and antibiotics.^12–14^ Indeed, while some vaccines have proved to be remarkably robust over time,^15–19^ others require continuous innovation.^12,20–27^ It logically follows that the scientific community must continually innovate to ensure that pathogens do not gain the upper hand. Vaccines efficiently exploit the immune system's natural capacity to recognize and establish long-term defenses against the unique markers of disease-causing agents.^28^ To successfully achieve protective immunity, a vaccine must include antigens that are either derived directly from the pathogen or introduced in the form of synthetic mimics.^29,30^ Generally, antigens include any substances able to induce a specific immune response or to be recognized by products of an immune response (such as antibodies).^31^ They include proteins, carbohydrates, nucleic acids, lipids, and haptens.^32^ The expansive natural diversity of antigens provides researchers who are actively searching for new leads with a vast pool of perspective immunostimulating agents. Carbohydrates are particularly attractive in this regard owing to the multitude of constituent monosaccharide building blocks, their diversity and complexity, and finally their ubiquity in nature.^33,34^ These biopolymers decorate the surface of almost^35–37^ every natural cell (prokaryotic and eukaryotic),^38^ where they are key mediators in essential recognition processes.^39,40^ The following cases are instructive to illuminate the societal benefits that may be gleaned from pursuing carbohydrate-based approaches: In bacterial cells, unique capsular polysaccharides (CPS) are common primary virulence factors, but they do not have easily targetable proteins on their surfaces (Fig. 1).^40^

Designing bespoke vaccines to emulate bacterial CPS^41^ can therefore lead to highly serotype-specific antibodies that precisely neutralize the desired pathogen.^42–44^ Moreover, numerous types of tumor cells display aberrant glycosylation patterns (tumor-associated carbohydrate antigens or TACAs) on their surfaces that aid detection.^45–47^ These specific patterns have inspired the development of carbohydrate-based therapeutic cancer vaccines that are designed to activate the immune system towards these unique signatures.^48–50^

Importantly, carbohydrate cellular fingerprints extend beyond these scenarios to encompass other chronic conditions such as neurodegenerative diseases^51^ and autoimmune disorders,^52,53^ among others.^54,55^ That said, the design and conception of functional carbohydrate-based vaccines remains a multifaceted challenge and general solutions remain elusive.^56,57^ As well as masking pathogenic proteins to enable immune evasion/infection, glycans are widely presented on human cells and engage in a multitude of functions.^58–60^ Consequently, eliciting sufficient immunogenicity poses a significant obstacle for glycan-based vaccines.^61–64^ The target glycan epitope is required to be distinguishable from sugars on healthy human cells to minimise the risk of autoimmune reactions.^56,65^ Simultaneously, the antigen must be sufficiently similar to the natural epitope to trigger the production of antibodies capable of recognizing the original structure,^60,66–68^*i.e.* cross-reactive antibodies.^69^

This requirement for structural similarity renders site selective fluorination an appealing molecular editing strategy.^70,71^ A combination of modest steric footprint and high electronegativity offers an opportunity to mimic specific hydroxyl groups on the glycan (OH → F),^71,72^ potentially enhancing the immune response without introducing unacceptable structural alterations. Vaccines based on fluorinated glycan antigens, which are not encountered in biology, offer a tempting opportunity to boost immunogenicity to trigger a stronger specific immune response with the formation of cross-reactive long-lasting antibodies. In this short Perspective, key considerations in vaccine development are presented for a non-specialist audience, and set in a wider discussion of the major milestones in the advent and development of fluorinated glycan vaccines leads. It is hoped that this will foster interest and discussion in this emerging area of targeted immunotherapy.

## 2. Vaccines and the immune system

From the perspective of function, vaccines are designed to train the adaptive immune system to recognize and eliminate a specific pathogen or cell group swiftly and effectively. The adaptive immune response is mediated by B cells that produce antibodies (humoral immunity) and by T cells (cellular immunity).^73^ Almost all vaccines in common use are thought to mainly confer protection through the formation of antibodies.^74^

**Fig. 2:**
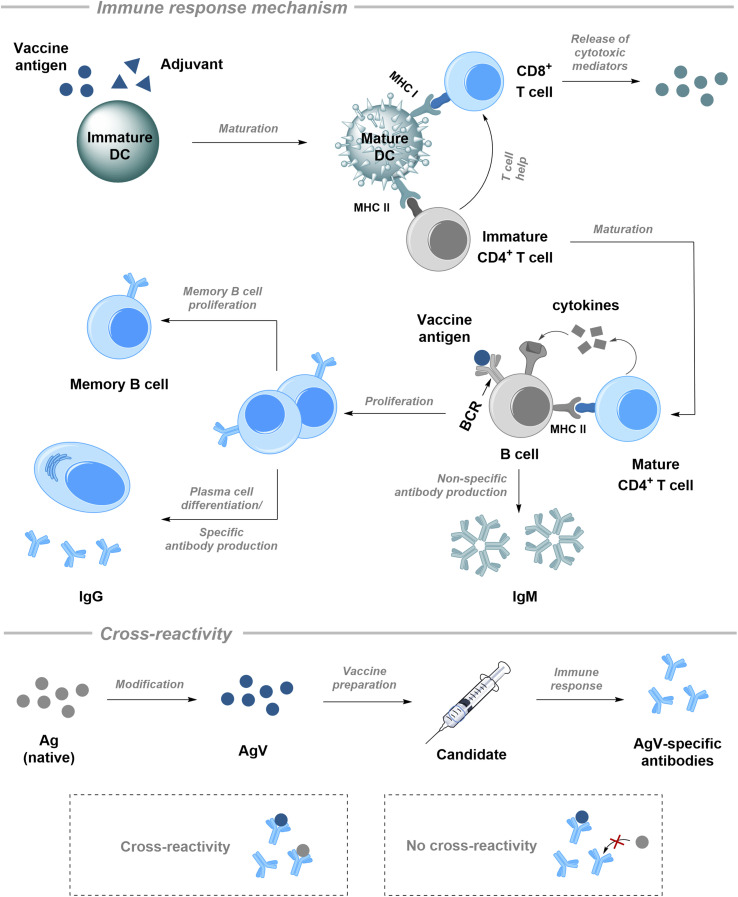
Simplified representation of the adaptive immune response to vaccine administration and the concept of cross-reactivity. Top: B cell can be activated without Th cell assistance (T cell-independently) *via* recognition of vaccine antigen to produce IgM or with the help of previously matured Th cell (T cell-dependently) leading to proliferation and IgG and long-term immunological memory. Bottom: modified antigen AgV induces formation of AgV-specific antibodies, which might recognize the original Ag, thus, be cross-reactive, or be exclusively specific for AgV – not cross-reactive – therefore, providing no protection against the original threatening epitope. DC = dendritic cell; MHC = major histocompatibility complex.

Following administration of a vaccine formulation, antigen-presenting cells (APCs), primarily dendritic cells (DCs), recognise “danger signals” such as those represented by adjuvants (components of vaccines that trigger a non-specific immune response; please see Section 3.1) (Fig. 2, top).^75–79^ This process triggers a sequence of events beginning with the maturation of DCs. Within the lymph nodes, mature DCs present processed antigen fragments on both major histocompatibility complex (MHC) class I and class II to naïve T cells. Antigen fragments on MHC I can activate CD8^+^ T cells that leads to the generation of cytotoxic mediators; these in turn can directly kill infected cells (cellular immunity). Antigen fragments on MHC II activate CD4^+^ T helper (Th) cells with subsequent proliferation and secretion of cytokines that drive the immune response toward either a humoral (antibody-driven) or cellular (cytotoxic-driven) profile.

For protective antibody production, B cell activation is crucial.^73^ Vaccine antigens can trigger a T cell-independent response by binding directly to B cell receptors (BCRs) present on the B cell surface. This leads to the production of less specific IgM-antibodies. To enable a more robust immune response, B cells require T cell help, *i.e.*, a T cell-dependent immune response. This is the basis for glycoconjugate vaccines, where a polysaccharide antigen is chemically linked to a protein carrier (please see Section 3.1),^80,81^ and allows B cells that recognize the polysaccharide to internalize the entire conjugate and process the protein carrier.^82^ Subsequently, they migrate to the border between the B cell and T cell zones in lymphoid tissue and present it on MHC II to previously activated, antigen-specific Th cells. There, if an activated Th cell recognizes the MHC II complex on the B cell, a cognate interaction occurs. This entire coordinated cascade results in B cell proliferation, generation of memory B cells, differentiation into antibody-secreting plasma cells, and antibody class switching: from less specific IgM to high-affinity, specific IgG; that dramatically changes the outcome of vaccination.

IgM is a fast primary responder,^83,84^ which is produced within the first days after the initial exposure. Its large, pentameric structure is ideally suited to bind multiple pathogens at once, agglutinating them for easier clearance by phagocytes.^85^ However it cannot provide a specific reaction. The biological half-life of IgM in the bloodstream is only about 5 to 7 days,^83,86^ which means its concentration drops rapidly after vaccination. For lasting immunological protection that effective vaccines must provide, IgG plays an important role.^87,88^ These antibodies take longer to be produced, but react with high specificity to the selected antigens during subsequent exposures (prophylactic effect) and provide the long-term immunological memory that is sought.^88,89^ It is important to stress, however, that is not sufficient to simply generate IgG antibodies; they must be cross-reactive. Cross-reactivity occurs when an antibody that was generated in response to a specific antigen (*e.g.* from a vaccine) also recognises and binds to a different, but structurally similar, antigen.^90,91^ This phenomenon has been successfully capitalized on through vaccine design, which enables vaccines to be effective against various bacterial or cell subtypes (serogroups/serotypes) and makes structural alterations of antigens possible.^92^ For example, a native pathogen antigen Ag undergoes modifications to give a new vaccine component AgV (Fig. 2, bottom). Antibodies raised after this vaccine injection will be specific for the modified AgV, but they are also required to recognize initial Ag from the target pathogen. Cross-reactivity makes this possible: with proper AgV design, the antibodies raised will recognize the native Ag as well and attack the pathogen, thereby affording protection to the individual.

## 3. Carbohydrate-based vaccines: the immune response and key components

**Fig. 3:**
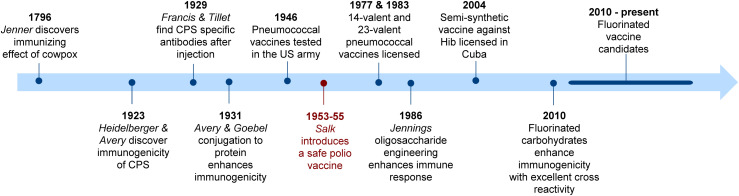
Timeline showing selected milestones in the field of carbohydrate-based vaccines.^47,56,93,96,104–106^

Carbohydrate-based vaccines have a venerable history that has its roots in the seminal discovery by Avery and Heidelberger in 1923, that the CPS of *Streptococcus pneumoniae* was immunogenic (Fig. 3).^93,94^ This key milestone led to the development of the first carbohydrate-based vaccine against pneumococcal disease.^95,96^ With the advent of antibiotics, the development of carbohydrate-based vaccines slowed, but the emergence of antibiotic resistance led to renewed interest in this field of research. To date, several carbohydrate-based vaccines have been licensed and are credited with having saved millions of lives. Among them are multivalent vaccines against *Streptococcus pneumoniae*, *Neisseria meningitidis*, and *Haemophilus influenzae* type b.^56^

### 3.1. Immune response

Soon after the establishment of native CPS based vaccines, it was observed that for certain groups in the population, the elicited immune response was not effective.^97^ This was especially problematic in patients under two years old. Only short-term IgM antibodies were elicited and no long-term protection was achieved. Carbohydrates (pathogen- or tumor-associated) tend to be poorly immunogenic due to the presence of similar or even identical antigens on the human cell surface.^61,64^ Although these glycans usually have lower density or are expressed during early development stages in healthy individuals, the difference is not significant enough to trigger a robust immune response.

#### 3.1.1. Carrier proteins

**Fig. 4:**
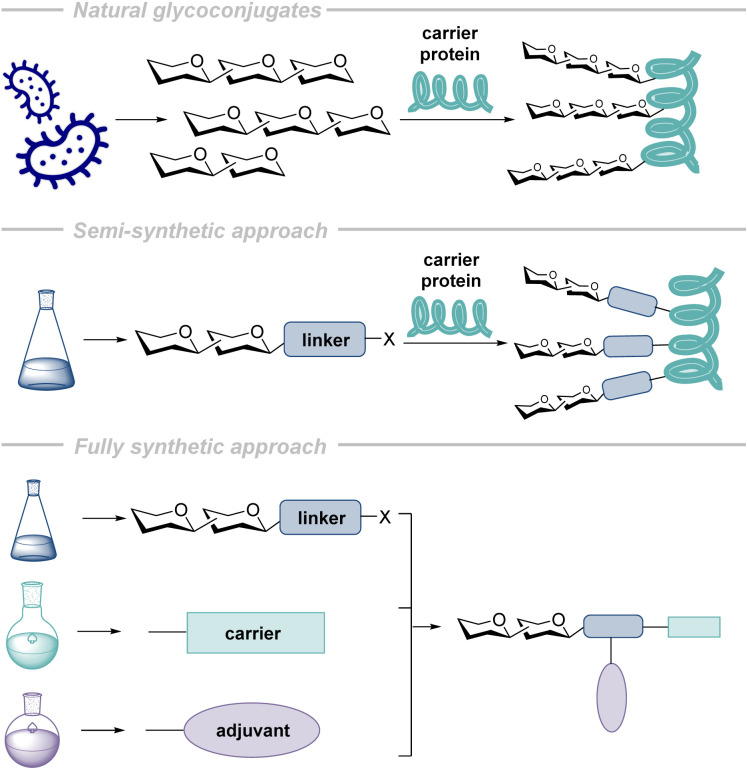
Schematic representations of the three approaches to glycan-based vaccine synthesis: the natural approach (all components are naturally derived), the semi-synthetic approach (certain components are naturally derived, while others are synthetic), and the fully synthetic approach (all components are synthetic).

A major advance in addressing the poor immunogenicity inherent to glycans has been to couple the glycan to a carrier protein to induce more robust immune responses.^98–104^ Carrier proteins increase the immunogenicity of the vaccine as they can initiate a T-cell-dependent immune response when they are processed by APCs and subsequently presented on MHC II to Th cells.^107,108^ As long as the original APC (B cell) has bound to the vaccine *via* the glycan epitope having been conjugated to the protein, T-cell assistance is associated with this specific sugar. Carrier proteins can be derived from pathogens like diphtheria toxin mutant (CRM_197_),^109^ tetanus toxoid (TT),^109^ diphtheria toxoid (DT),^110^ meningococcal outer membrane protein complex (OMPC),^109^*Hemophilus influenzae* protein,^109^ and *Pseudomonas aeruginosa* exotoxin A (rEPA).^111^ These proteins can also originate from non-pathogenic (and therefore often more easily accessible) sources like bovine serum albumin (BSA)^112^ and keyhole limpet hemocyanin (KLH).^113^ Glycoconjugate vaccines can therefore be grouped into three different types as summarized in Fig. 4: (1) natural, (2) semi-synthetic, and (3) fully synthetic glycoconjugates.^98^

**Fig. 5:**
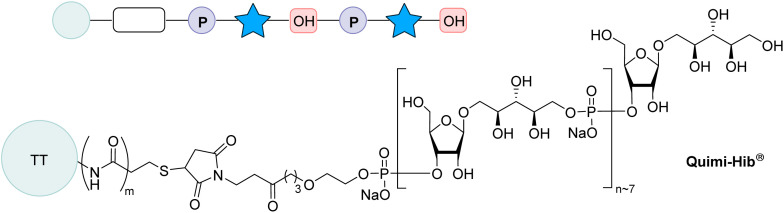
Structure of the semi-synthetic vaccine included in the Cuban and South American vaccination schedule.^106^

In the case of natural glycoconjugates, both components are derived from natural sources such as microbial cultures.^114^ Despite their efficacy against the corresponding pathogens, major issues are associated with this manufacturing method and include complicated and expensive purification procedures still leading to batch-to-batch variability, the presence of cell-derived impurities, and uncontrolled and irreproducible protein conjugation chemistry.^56^ In comparison, synthetically derived carbohydrates are pure, well-defined and can be equipped with a synthetic linker to facilitate controlled coupling to the carrier protein. This type of glycoconjugate is termed semi-synthetic.^115^ In 2004, a Cuban research team published the first industrial scale synthesis of a semi-synthetic carbohydrate-based vaccine (Quimi-Hib®) that was later included in the Cuban and South American vaccination schedule to address *Haemophilus influenzae* type b (Hib) (Fig. 5).^106^

It is important to note that the protein in this approach is essential to ensure a robust immune response, but it suffers from some of the challenges associated with the natural approach mentioned above, such as inconsistent conjugation results.

#### 3.1.2. Adjuvants

In contrast to the approaches mentioned above, fully-synthetic glycoconjugates are free of many of the drawbacks associated with using naturally-sourced components.^115^ These therapeutics are composed of the antigen glycostructure coupled to a synthetic carrier (such as proteins or nanoparticles^116^) and combined with adjuvants to ensure a robust immune response. Adjuvants are agents that non-specifically augment the immune response to a co-administered antigen, without being the primary target of the adaptive immune response. The major adjuvant groups include: alum-based, bacteria-derived, liposome adjuvants, and adjuvant emulsions.^117,118^ In approved glycoconjugate vaccines, as well as in many vaccine candidates, adjuvants are used in the combination with proteins to achieve the desired immune response.

#### 3.1.3. Other approaches

The field of fully synthetic vaccines is relatively new and there are currently no candidates in clinical trials that the authors are aware of. A conspicuous challenge is imitating the immune response generated by the carrier protein.^68^ Recent findings also indicate that zwitterionic polysaccharides can induce robust antibody production without conjugation to a carrier. Alteration of the 3D structure of the vaccine (*e.g.*, the organisation of glycoclusters on dendrimeric structures) has led to positive outcomes as well.^119^ The modularity of this approach enables combinations of different carbohydrate antigens to be leveraged in the pursuit of multivalent vaccines against different serogroups of the same bacterium. A prominent example is a 20-valent pneumococcal vaccine that provides protection against 20 serotypes of *Streptococcus pneumoniae*.^120^ However, it is important to emphasise that a limiting factor in the development of synthetic glycoconjugate vaccines is the chemical synthesis of carbohydrates; this remains challenging and time-consuming. One method to simplify glycan synthesis is automated glycan assembly (AGA),^121^ which was formalized by the Seeberger group in 2001 ^122^ and has undergone rapid growth in recent years.^123–128^ Using pre-functionalized building blocks, AGA allows for a “plug and play” approach to generate the desired oligosaccharides in a rapid and efficient way. Contemporary AGA workflows can be conducted on commercial synthesizers (*e.g.*, the Glyconeer 2.1® instrument)^129^ to produce glycans that are directly amenable to subsequent conjugation.

#### 3.1.4. Structure modification

**Fig. 6:**
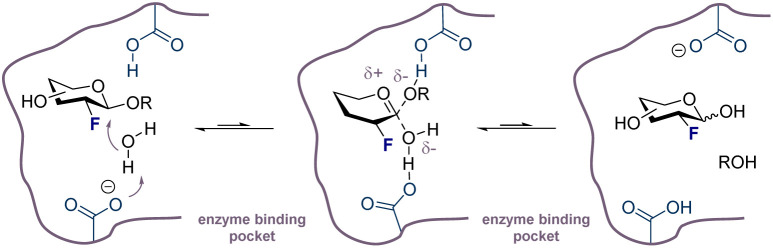
Simplified representation of a fluorinated glycan in a hydrolase binding pocket. Fluorine destabilizes the transition state and plays an active role in enhancing hydrolytic stability.^139^

Strategically introducing non-native antigens is an expansive approach to overcome immune tolerance and induce robust immune responses. However, these antibodies must closely simulate recognition of the original target antigens. In 1986, Jennings and co-workers first demonstrated that the chemical modification of a carbohydrate antigen enhanced antigen immunogenicity, but these early examples were limited by a low cross-reactivity of the elicited antibodies with their native structures.^130^ This discovery remains a foundational milestone for the field, and its impact is reflected in the chemically modified carbohydrates that have already reached clinical trials. For example, a vaccine targeting small cell lung cancer using *N*-propionyl-modified α-(2→8)-linked polysialic acid^131^ and the melanoma vaccines with GD2- and GD3-lactone derivatives are powerful exemplars.^132^ Given its success in medicinal chemistry, it logically follows that strategic fluorination of antigenic glycans presents a promising direction for native glycan modification. Fluorination of glycostructures is a potentially powerful editing approach that allows a sugar to be subtly modified in a minimally invasive manner whilst offering the possibility to influence physicochemical parameters and immunogenicity. This is largely grounded in the unique properties of the fluorine atom (small size similar to hydrogen and high electronegativity) making it a suitable bioisostere of the H-atom and OH-group.^71,72^ Selective bioisosteric substitution with the F-atom has been successfully integrated into glycoengineering strategies for studying antibody behavior.^133,134^ Fluorinated glycostructures have been shown to be more hydrolytically stable, which have positive consequences for bioavailability: enzymatic degradation is inhibited significantly by destabilisation of the transition state. The effect can be convincingly observed when the C(sp^3^)–F is incorporated into different positions of the sugar, with the largest stability benefits conferred when fluorine is placed next to the anomeric centre (Fig. 6).^134–138^ The combination of these factors renders fluorinated glycans potentially valuable from the perspective of designing next generation of carbohydrate-based vaccines.

An additional aspect of C2 fluorination that is particularly appealing in synthetic planning is the ability of this small substituent to regulate the selectivity of glycosylation reactions. Not only does this make chemical glycosylation predictable and efficient,^140–144^ the ^19^F nucleus facilitates analysis by NMR. The current field of fluorosugar research is multifaceted; it includes a diversity of synthetic methods for fluorine incorporation,^145–147^ the use of fluorine as a directing group in glycosylation,^140–144^ and the practical application of fluorosugars in bio-medicine and chemical biology.^148–155^ In developing lines of research, fluorination can simplify the synthesis of glycan building blocks, rendering them more suitable for automated glycan assembly. In 2021, the Delbianco group presented an automated approach to fluorinated Lewis type 2 antigens.^156^ In 2022, the Gilmour group disclosed an automated synthesis of HIV-relevant fluorinated high mannose structures using the Glyconeer 2.1® synthesizer.^157^ Furthermore, in 2026, the same platform was used to generate fluorinated frameshifts of *Klebsiella pneumoniae* antigens to investigate how antigen frameshifts affected binding to the lectin Concanavalin A.^158^ These representative examples serve to illustrate the levels of complexity that can be generated in F-glycan mimetics, and the opportunities that this technology affords in vaccine development.

## 4. Fluorinated carbohydrate vaccines

### 4.1. Prophylactic vaccines

Prophylactic carbohydrate-based vaccines are administered prior to an initial infection and protect against future infection by the target pathogen. Lacking easily targetable proteins on their surfaces, certain bacteria rely on their unique CPS to infect a host, evade immune responses, and cause disease. This capsule physically shields the bacterium, enabling it to evade initial immune detection by masking underlying antigens. Vaccines targeting this capsule induce highly specific antibodies that can neutralise the pathogen with remarkable precision. Research has consistently shown that antibodies directed at polysaccharides are highly beneficial in preventing bacterial infections.^159^ Consequently, vaccines based on these capsular polysaccharides, in both monovalent and multivalent forms, have been approved for use against *Streptococcus pneumoniae*, *Neisseria meningitidis*, *Haemophilus influenzae* type b (Hib), and *Salmonella typhi*.^160^ Regrettably, this does not guarantee permanent protection against these infections, and new and modified vaccines are required.

#### 4.1.1. Neisseria meningitides

*Neisseria meningitidis* (meningococcus) is a Gram-negative bacterium known to be a causative agent of meningococcal meningitis and meningococcal septicemia.^161^ Sialic acids play a significant role in the biology and pathogenicity of *N. meningitidis*, shaping its lipooligosaccharide (LOS) structure. Sialylation of LOS makes the bacterium appear more “host-like”, reducing its recognition by the immune system. *N. meningitidis* serogroup B CPS contains α-(2,8)-linked *N*-acetylneuraminic acid (Neu5Ac),^162^ a structure also prevalent in human neuronal glycans.^163^ Due to this molecular mimicry, the epitope is poorly immunogenic and carries risks of inducing autoimmunity. In contrast, the α-(2,9)-linked sialyl epitope is uniquely bacterial and absent from human tissues, making it an attractive target for vaccine design.^164,165^

**Fig. 7:**
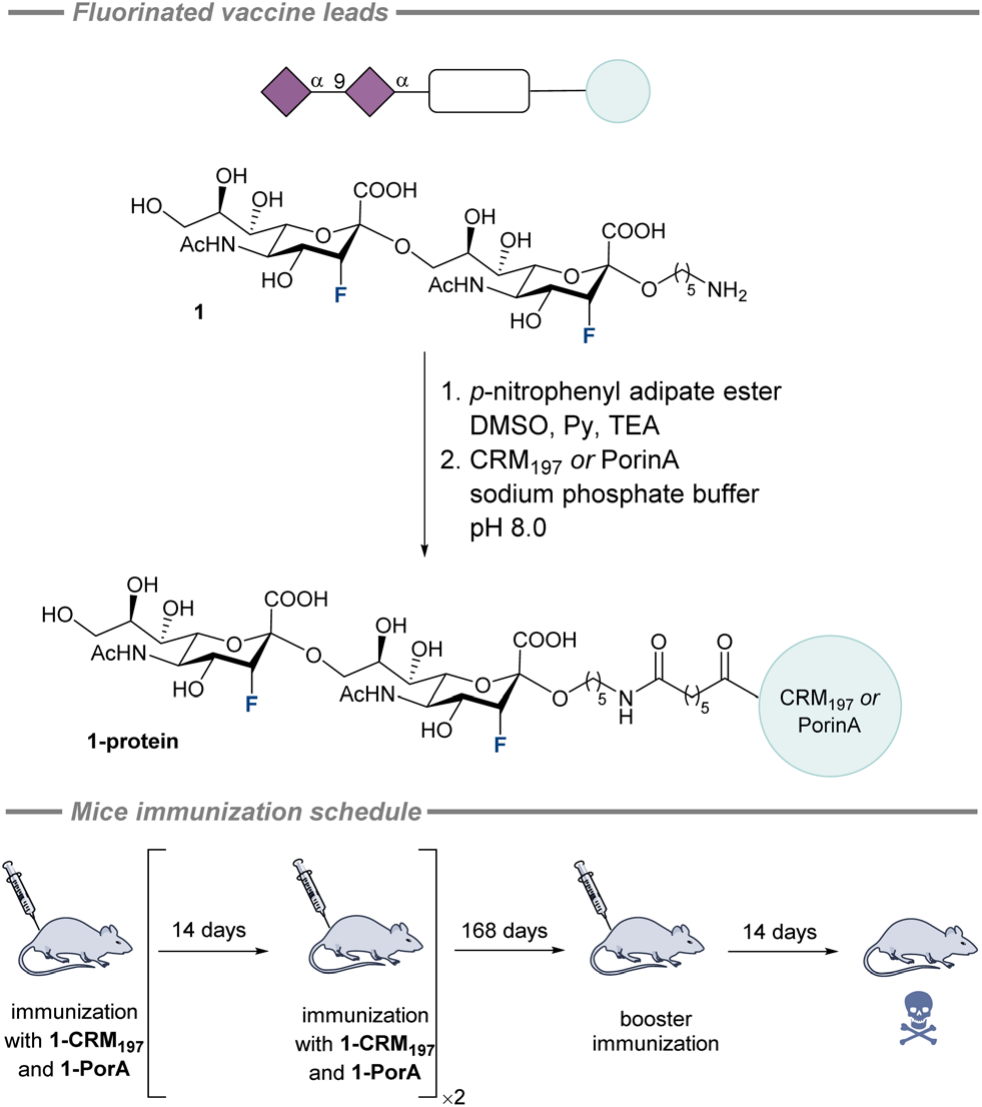
Fluorinated sialic acid vaccine lead against meningitis B and C reported by the Gilmour and Seeberger groups and the schedule of mice immunization with candidates 1-CRM_197_ and 1-PorA.^166^

This observation inspired the work of the Gilmour and Seeberger groups on a fluorinated sialic acid vaccine lead against meningitis B and C.^166^ The shortest immunogenic unit was described as a disaccharide^44^ informing the vaccine design which was a fluorinated disialoside (1) with a linker for protein coupling at the reducing end (Fig. 7, top). Fluorine was strategically introduced at the C3 position adjacent to the anomeric centre since C3 fluorinated sialic acid derivatives do not undergo elimination during glycosylation and the fluorine also acts as a directing group affording mostly α-product.^167,168^ In addition, the fluorine also functions as a practical and sensitive NMR-active nucleus that expedites structural analysis. Following global deprotection, the glycan was conjugated *via* a *p*-nitrophenyl adipate ester to two different carrier proteins: CRM_197_ and PorA. To investigate the influence of these candidates on the immune response, the study employed either aluminium hydroxide adjuvant (Alum) or Freund's Adjuvant. For immunological studies, four groups of six of C57BL/6 mice were immunized in accordance with the schedule (Fig. 7, bottom). Given the expected increase in immunogenicity from fluorination, a much smaller amount of the conjugate vaccine was used.

All tested glycoconjugates successfully activated the immune system in a T-cell-dependent manner, and this was reflected in the similar long-term IgG responses observed. The highest level of antibodies was achieved on day 70, after which the levels decreased. The booster immunization triggered the rise of IgG levels back to previous levels, revealing production of memory B cells. IgG_1_ isotype antigens were predominantly formed, IgG_2_, and IgG_3_ were indicated in lower amounts. Among those, IgG_1_ and IgG_3_ can effectively bind and activate complement. All glycoconjugates demonstrated specific binding to *N. meningitidis* C CPS and to whole bacteria. *N. meningitidis* B CPS was specifically recognized only by antibodies induced by 1-PorA containing conjugates. Antibodies induced by 1-PorA-Alum were found to be protective against both *N. meningitidis* B and C and 1-CRM_197_-Alum displayed the highest level of protection.

#### 4.1.2. Leishmania donovani

*Leishmania donovani* is a parasitic protozoan that causes visceral leishmaniasis, also known as kala-azar or black fever.^169^ It is a life-threatening condition because it primarily targets the internal organs, most notably the liver, spleen, and bone marrow.

**Fig. 8:**
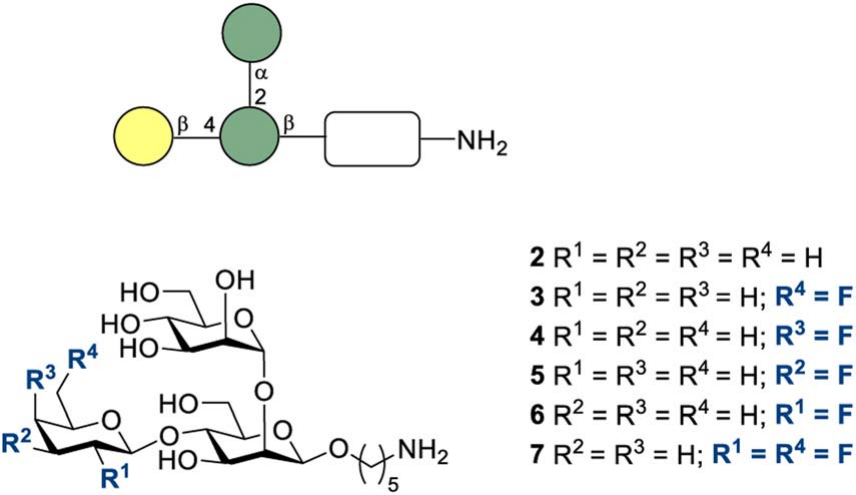
*L. donovani* trisaccharides with different fluorination patterns of the galactose unit synthesized by Hoffmann-Röder and co-workers.^170^

In 2018, Hoffmann-Röder and co-workers reported the synthesis of fluorinated analogues of the trisaccharide *L. donovani* CPS component 2 (Fig. 8).^170^ The authors focussed particular attention on the galactose subunit, which was systematically fluorinated. The synthetic strategy enabled the target disaccharides to be coupled to the terminal mannose subunit to furnish the desired trisaccharides with the NH_2_-linker (3–7). In so doing, modified epitopes potentially suitable for the development of diagnostic tools and synthetic anti-leishmanial vaccines were developed. According to the authors, work in the aforementioned direction is in progress.

#### 4.1.3. Streptococcus pneumoniae

*Streptococcus pneumoniae* (pneumococcus) is a Gram-positive bacterium with a dual nature: it's a common colonizer of the upper respiratory tract, but also a major human pathogen.^171^ Consequently, *S. pneumoniae* presents a threat to infants, immunocompromised patients, and the elderly. The impact of the bacterium, though reduced, remains significant due to its ability to adapt to antibiotics and serotype replacement.^171^*S. pneumoniae* serotype 8 (ST8) is now one of the top-ranking serotypes causing invasive pneumococcal diseases as it is only partially covered by the glycoconjugate vaccines in use (present in PPSV23 and PCV-20 and absent in PCV-7, PCV-13, and PCV-15).^120,172^ This makes the ST8 capsular polysaccharide an important target for vaccine development.

**Fig. 9:**
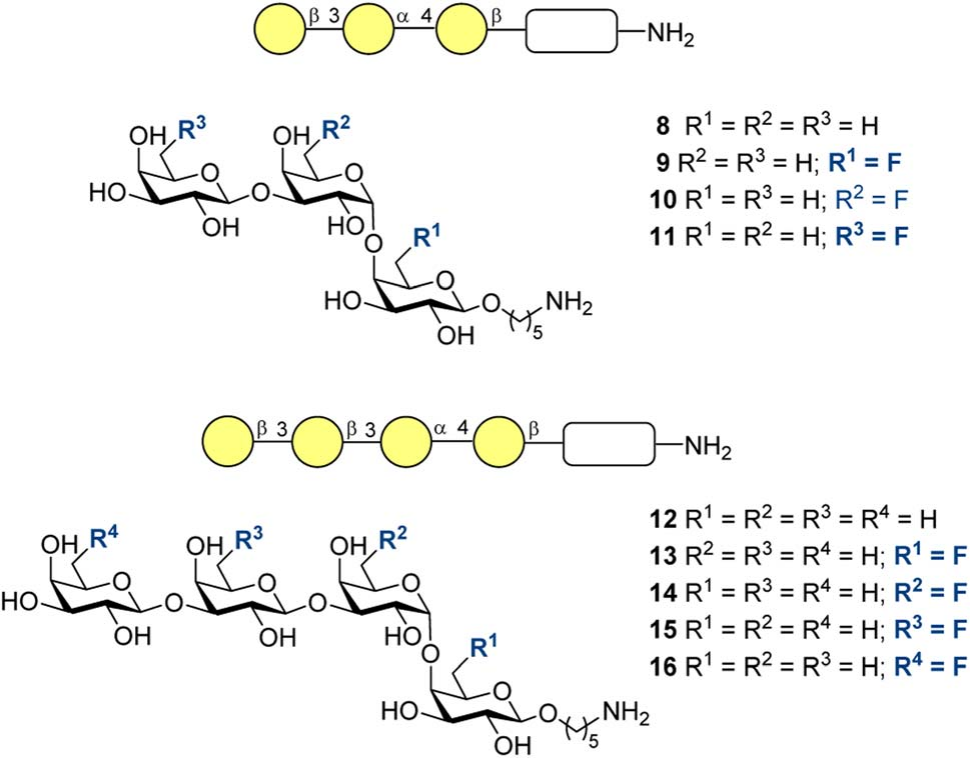
*S. pneumoniae* ST8 tri- and tetrasaccharides with different fluorination patterns at the 6-position of the galactose units synthesized by Hoffmann-Röder and co-workers.^174^

The minimal protective epitope of ST8, which appeared to be a trisaccharide, was identified in 2017 by Schumann and co-workers.^173^ It was found that by conjugating it to a glucose moiety, the relatively low immunogenicity can be enhanced. An important study by Hoffmann-Röder and co-authors validated synthetic routes to fluoro-substituted glycans 8–16 with a linker at the reducing end for subsequent conjugation steps (Fig. 9).^174^ The 6-position was chosen as the most exposed site and each of the molecules obtained contained only one fluorinated unit for the sake of subsequent systematic investigation of the heteroatom impact.

**Fig. 10:**
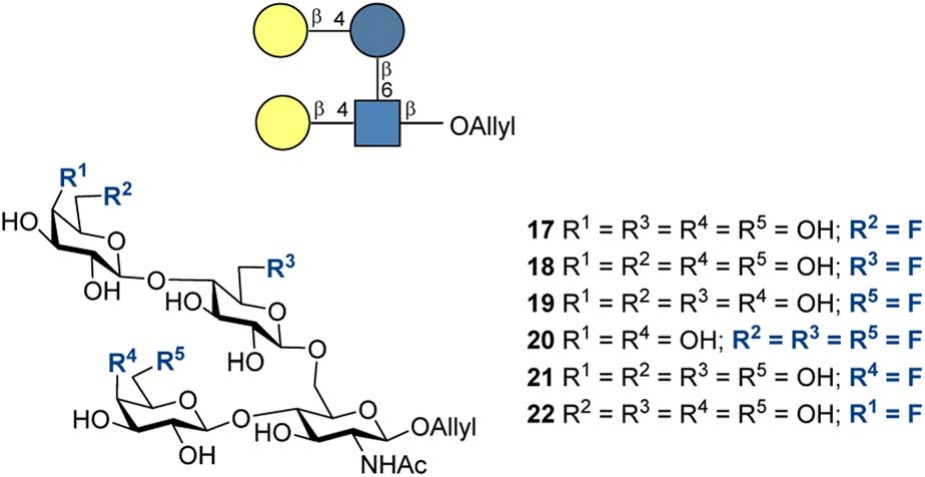
*S. pneumoniae* ST14 tetrasaccharides with different fluorination patterns synthesized by Hoffmann-Röder and co-workers.^175^

In 2026, the same group described a synthetic approach to the minimal protective antigenic structure of CPS of *S. pneumoniae* ST14 (Fig. 10),^175^ which has found application as a component of several vaccines in current use.^176^ The authors prepared tetrasaccharides 17–22 with allyl groups appended at C1: this is a convenient handle for conjugation to a carrier protein. These materials will certainly be valuable in determining the influence of fluorination on the subsequent antibody production and vaccine design.

#### 4.1.4. Summary

From these selected case studies, it is evident that fluoro-glycans hold great promise in the conception of vaccine leads against bacteria, but that the field is at an early stage. Whilst the sialic acid-based vaccine lead against meningitis B and C demonstrates convincing proof of concept in bolstering immunogenicity, stability, and the induced antibodies' cross-reactivity, there is much to be done. Further advancement of vaccine candidates will require a concerted, interdisciplinary approach that includes preclinical testing, scalable manufacturing, and compelling evidence that they are more effective and safer than the current vaccines. This need to innovate in opening up new areas of glycomimetic chemical space is also evident from how a popular commercial vaccine against pneumococcus – PCV-7 and PCV-13 – having immunized millions of people was futile against ST8 and supported the rise of the ST8 epitope.

### 4.2. Therapeutic vaccines

In the arena of therapeutic carbohydrate-based vaccines, emphasis has been heavily concentrated on the aberrant glycosylation patterns of malignant cells.^8,65^ Tumor-associated carbohydrate antigens (TACAs) are antigens, which are over- or uniquely expressed on the tumor cell surface.^45,75,179^ TACAs arise from alterations in sugar chain structure, including truncation, sequence changes, or excessive sialylation of glycolipids and glycoproteins. Abnormal glycosylation is shown to be linked to key stages of cancer progression, such as invasion and metastasis. Since many TACAs are predominantly expressed during cancer development and are largely absent from normal adult tissues, they represent promising targets for therapeutic cancer vaccines.^180,181^ A wide range of TACAs have been characterized, including: the mucin related (*O*-linked) GalNAc (Tn), sialyl Tn (STn), Thomsen–Friedenreich (TF), sialyl TF; the glycosphingolipids Globo H, stage-specific embryonic antigen-3 (SSEA-3); the gangliosides GM2, GD2, GD3, fucosyl GM1; polysialic acid (PSA).^182,183^

**Fig. 11:**
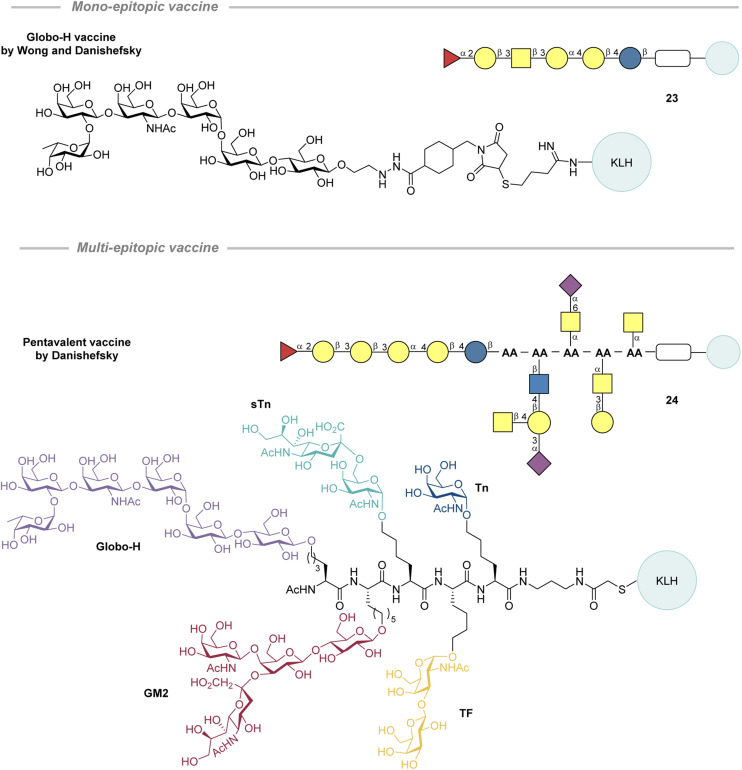
Therapeutic carbohydrate-based vaccine leads based on TACAs: mono-epitopic Globo H vaccine by Wong, Danishefsky and co-workers and multi-epitopic pentavalent vaccine by Danishefsky and co-workers; KLH = keyhole limpet hemocyanin.^177,178^

Carbohydrate-based cancer vaccines can be grouped into three different categories: (1) mono-epitopic, (2) mono-epitopic cluster (presents multiple copies of a single epitope), and (3) multi-epitopic vaccines.^184^ A mono-epitopic vaccine of Globo-H 23, an unnatural TACA, was developed by Wong, Danishefsky, and co-workers (Fig. 11, top).^178^ Overall, five different total syntheses have been reported, of which an enzymatic sequence proved the most cost effective, with a yield of over 80% over only two steps. In Phase I trials, a synthetic Globo-H-KLH conjugate was tested and the study confirmed the immunogenicity and safety of the vaccine candidate, which has now moved to Phase II/III trials. A multi-epitopic, unimolecular, and pentavalent vaccine 24 that targets Globo-H, STn, Tn, TF, and GM2 was developed by Danishefsky and co-workers (Fig. 11, bottom).^177^ The vaccine candidate showed induction of IgG and IgM antibodies against all five of the carbohydrate antigens. Unfortunately, these vaccine candidates suffer from non-specific, short-lived immune responses. To overcome this, considerable effort has been invested in increasing immunogenicity *via* sugar structure modification, especially fluorination (for more examples of non-fluorinative modifications please see Section 3.1.4).

#### 4.2.1. Thomsen-nouveau (Tn) antigen

The Thomsen-nouveau (Tn) antigen (25) is a truncated *O*-linked glycan bound to mucin proteins *via* serine or threonine. It consists of *N*-acetyl galactosamine.^185^ Tn is a known TACA and has been found to be present in up to 90% of cases across a spectrum of cancers.^186^

**Fig. 12:**
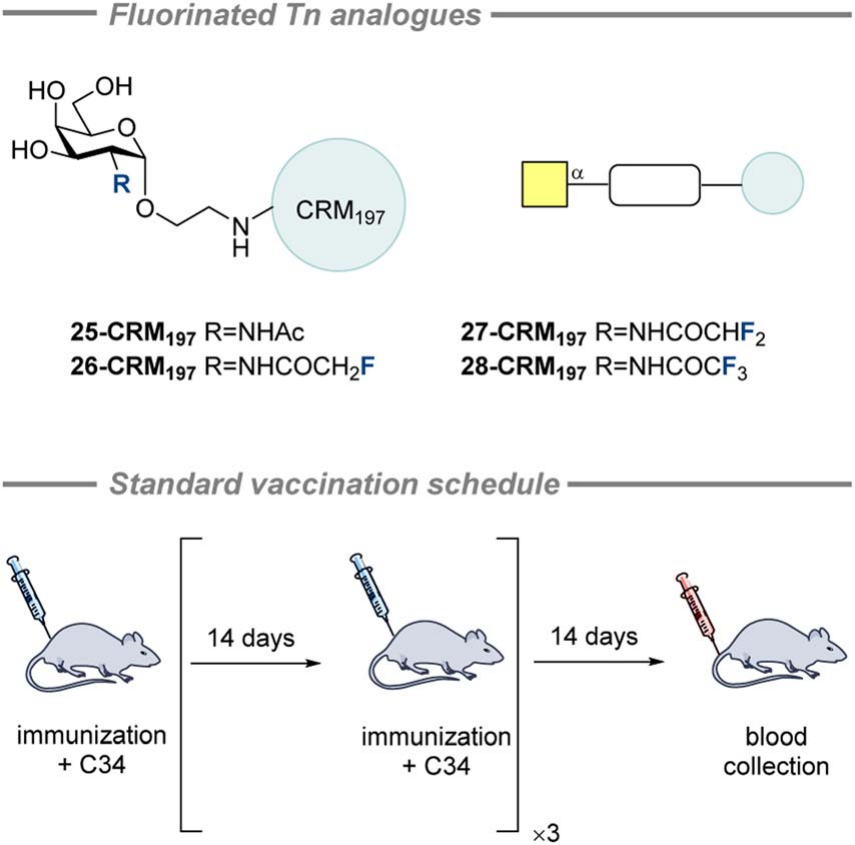
Fluorinated Tn analogues with modified *N*-acyl groups by Ye and co-workers and the standard immunization scheme applied by the Ye group.^187^

In 2016, Ye and co-workers synthesized and evaluated three fluorinated Tn analogues by modifying the *N*-acyl group (Fig. 12).^187^ Analogues with a monofluoromethyl group (26-CRM_197_), a difluoromethyl group (27-CRM_197_), and a trifluoromethyl group (28-CRM_197_) on the *N*-acyl moiety were prepared by reacting a 2-amino galactose derivative with the corresponding methyl ester of the fluorinated motif. The compounds were conjugated to CRM_197_ and combined with the C34 adjuvant for the immunization of mice in accordance with the schedule (Fig. 12, bottom). After 4 vaccinations, the anti-STn IgG antibody titer was greatest for 26-CRM_197_ (2.7 times greater than that of 25-CRM_197_). However, 27-CRM_197_ and 28-CRM_197_ led to a lower IgG titer than when vaccinating with 25-CRM_197_. Furthermore, flow cytometry revealed that the immunized (26-CRM_197_) mouse sera could recognise Tn in its native environment on cancer cells (MCF-7 and Jurkat) after the third vaccination. It was also found that vaccination with 26-CRM_197_ led to a higher proportion of IFN-γ producing splenocytes than for 25-CRM_197_, indicating a Th1 skewed response and that fluorination patterns offer a potential solution.

#### 4.2.2. Sialyl-Thomsen-nouveau (STn) antigen

**Fig. 13:**
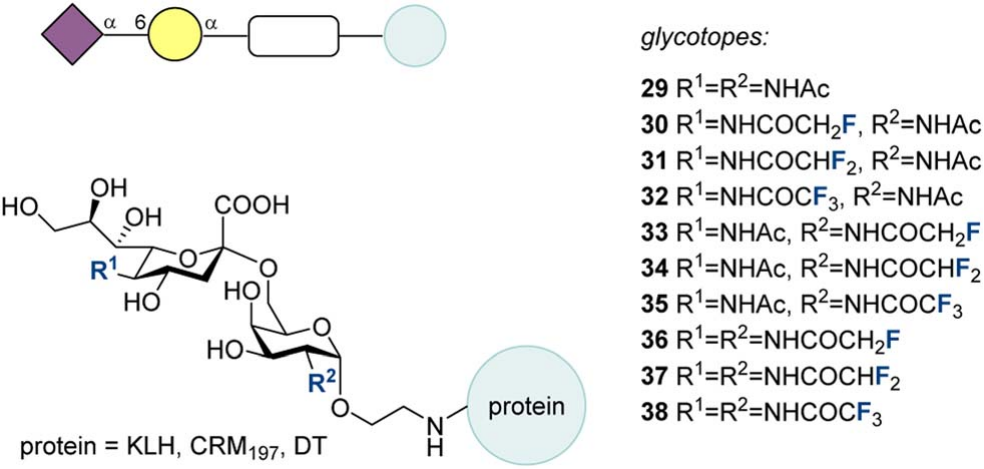
Fluorinated STn analogues synthesized by modifying the two amide groups of STn by the Ye group.^47,194^

The sialyl-Thomsen-nouveau (STn) (29, Fig. 13) antigen is a TACA associated with poor prognoses.^188^

This truncated *O*-antigen is capped by sialylation (of the Tn antigen) and linked to a protein *via* a serine or threonine residue at the reducing end. There has been interest in exploiting the STn antigen as a potential target for vaccination in oncology. One high-profile example was Theratope®, a conjugate of STn and KLH that was developed as a potential vaccine against metastatic breast cancer.^189^ In initial trials this candidate showed great promise with significant anti-STn IgG titer.^190^ However, in 2003, a phase III trial with 1028 patients showed that the vaccine didn't significantly prolong survival (23.1 months *vs.* 22.3 months for a control group).^191^ This unexpectedly poor performance was explained as possibly resulting from low STn expression on the tumors (they were not phenotyped), the trial not being long enough, or an inadequate immune response.^191,192^ This was despite the vaccine being administered after cyclophosphamide treatment to suppress regulatory T cells (Tregs)^193^ and with an adjuvant for the first vaccination to enhance the immune response.

In a bid to develop more immunogenic STn vaccine candidates, in 2010, Ye and co-workers synthesized a panel of STn analogues by modifying the two amide groups of STn (Fig. 13).^47^ Compounds 30–38 have the trifluoro-, difluoro- and monofluoromethyl groups incorporated into the amides. A variety of other amide modifications were investigated but here only those containing fluorine will be discussed. Enzyme-linked immunosorbent assay (ELISA) screening was used to identify promising potential vaccine candidates. The most promising candidates (*i.e.*, strongest binders) were conjugated to keyhole limpet hemocyanin (KLH) for *in vivo* immunological evaluation in mice. Important to note is that no adjuvants were used to enable a more robust comparison between glycan epitopes. After 4 vaccinations (Fig. 12, bottom), pooled sera were analyzed to measure the ani-STn IgG and IgM titers.

This revealed that the CF_3_ modifications (32-KLH, 33-KLH, 38-KLH) were detrimental in all positions. Modification of only the sialic acid amide (30-KLH, 31-KLH, 32-KLH) also offered no improvement over native STn. However, a monofluoromethyl amide on the galactose (33-KLH) or on both sugars (36-KLH) led to anti-STn IgG titers 2 times and 2.3 times higher than 1-KLH, respectively. A difluoromethyl amide was beneficial on the galactose moiety (34-KLH) resulting in anti-STn IgG titers 2 times higher than 1-KLH. This revealed that 33-KLH, 34-KLH, and 36-KLH have potential as vaccine candidates against STn, and that they may be able to overcome the problems observed when using native STn as a vaccine. The IgG/IgM ratio was found to be higher for the fluorinated compounds compared to the natural 30. The binding of these antibodies to STn in its natural environment was proven using flow cytometry to confirm binding to human cancer cells expressing STn. Specificity was also demonstrated by the diminished binding to STn deficient human cancer cells.

Candidates 34, 35, and 37 underwent further testing in 2017, with a murine tumor model using murine colon cancer cells.^195^ During a course of five bi-weekly vaccinations, after the penultimate vaccination, mice were injected with CT-26 cells in a tumor challenge study. Cyclophosphamide was given before the initial vaccination, which used Freund's complete adjuvant. Subsequent vaccinations utilized Freund's incomplete adjuvant. Of the three tested vaccines, 36-KLH showed a significant reduction in tumor burden in the lungs compared to other vaccines, including 29-KLH, and significantly extended the survival time.

With the promising activity of fluorinated 36 established, attention turned to investigating other carrier proteins, *e.g.*, CRM_197_.^196^ In 2019, Ye and co-workers synthesized 36-CRM_197_ and compared it to 29-CRM_197_ with (Freund's or C34) and without adjuvant.^194^ The findings showed that the anti-sera from the fluorinated vaccine were more effective at promoting cancer cell lysis by complement dependent cytotoxicity (CDC) and antibody dependent cellular cytotoxicity (ADCC) pathways. Furthermore, analysis of splenocytes revealed they produced interferon-gamma (IFN-γ) and interleukin 4 (IL-4) upon *in vitro* stimulation by the glycoconjugate, indicating a mixed Th1/Th2 response. This mixed cellular and humoral response was found to be stronger with splenocytes from 36-CRM_197_ vaccinated mice *versus* those vaccinated with 29-CRM_197_. Furthermore, the anti-STn IgG titers were higher for 36-CRM_197_ with and without adjuvant, confirming the fluorinated motif 36 yielded a multifaceted improvement to the immune response.

**Fig. 14:**
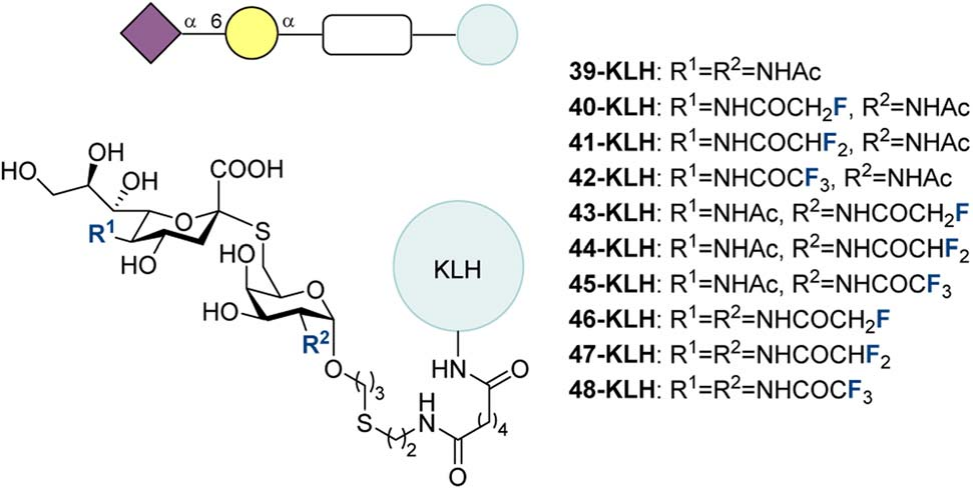
Fluorinated S-linked STn analogues synthesized by modifying the two amide groups of STn by the Ye group.^197^

With the intention of developing another panel of potential STn vaccine candidates with improved immunogenicity, in 2015, Ye and coworkers reported the synthesis and evaluation of fluorinated S-linked STn conjugates (Fig. 14).^197^ With the fluorination verified to improve the immune response to STn in previous work,^195^ the sulfur linkage was purported to contribute to the “non-self” nature of the glycan and to improve its stability, which has been demonstrated in literature.^198,199^

The previous conjugates of 29–38 with KLH were prepared by linking the glycan bearing an allyl group to the protein, first by oxidation to the aldehyde and then by reductive amination to couple the glycan to the protein.^195^

None of the S-linked STn conjugates resulted in higher anti-STn IgG titers than native STn (29-KLH). The lower anti-STn IgG titers for the S-linked conjugates were not a result of poor immunogenicity, but rather of poor cross reactivity of the antibodies with native STn. It was observed that fluorination could increase the anti-native STn IgG titers in mice compared to the non-fluorinated S-linked conjugate 39-KLH by improving the cross reactivity of the antibodies. The best performing S-linked conjugate was 46-KLH with two monofluoromethyl amides, though this still resulted in less than 10% of the titer seen for 29-KLH after 4 vaccinations without adjuvant.

Overall, these studies of modified STn conjugates indicate that fluorinated S-linked STn derivatives (as opposed to S-linked STn) could be promising vaccine candidates owing to their improved immunogenicity compared to 29. STn derivative 36 with two monofluoromethyl amides showed particularly promising behaviour.

#### 4.2.3. Microvesicles

**Fig. 15:**
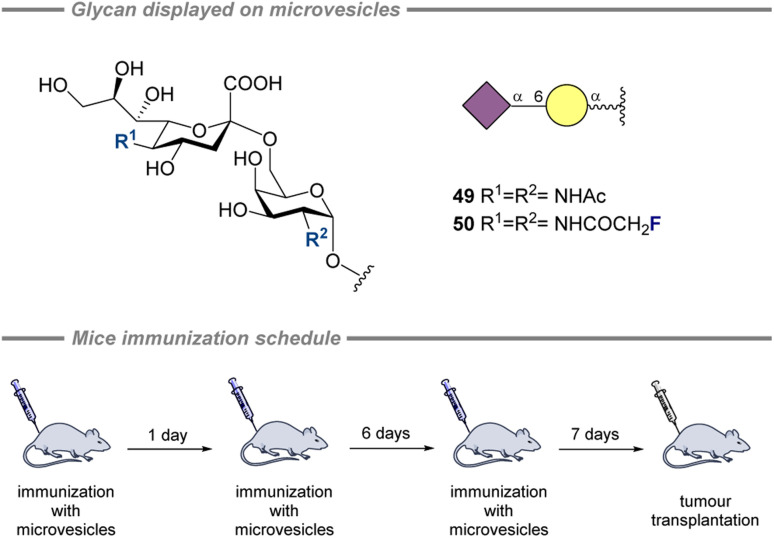
Fluorinated analogues of STn displayed on microvesicles and the immunization schedule.^200^

Microvesicles displaying fluorinated analogues of STn were shown to be potentially useful as cancer vaccine leads by Ye and coworkers in 2023 (Fig. 15).^200^ The microvesicles bearing modified STn (50) were produced by incubating cells with fluorinated carbohydrate precursors and isolating the resulting microvesicles. Mice had been immunized with the microvesicles prior to implantation of a tumor (Fig. 15, bottom). Immunization with 50 proved to be more effective at tumor growth inhibition compared to 49 (*i.e.*, CH_3_ to CH_2_F had a positive impact). Immunization with microvesicles also reduced metastasis compared to wild type microvesicles when mice were challenged with cancer cells. It was reported that one of the reasons for the benefits seen using the fluorinated STn (50) was an increased affinity to the major histocompatibility complex I (MHC-I).

#### 4.2.4. Thomsen–Friedenreich (TF) antigen

**Fig. 16:**
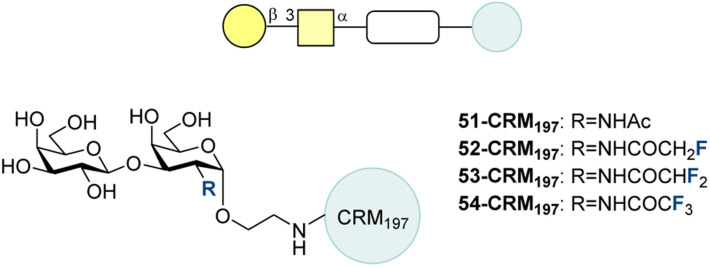
Fluorinated TF analogues synthesized by modifying the amide group of TF by the Ye group.^204^

The Thomsen–Friedenreich (TF) antigen is a disaccharide TACA commonly found in breast and prostate cancers.^201^ TF-KLH was explored as a potential vaccine, with the suite of leads structures including fluorinated derivatives.^202,203^ In 2016, Li, Ye and co-workers synthesized the TF antigen (51) as well as modifications of the *N*-acetyl group: monofluoromethyl (52), difluoromethyl (53) and trifluoromethyl (54) (Fig. 16).^204^ These were conjugated to CRM197 and used to vaccinate mice with C34 as an adjuvant. After 4 vaccinations, 52-CRM_197_ led to the highest anti-51 IgG titer (2.5 times greater than 51-CRM_197_). The trifluoromethyl derivative 54-CRM_197_ led to anti-TF IgG titers 1.6 times larger than 51-CRM_197_. However, the difluoromethyl derivative 53-CRM_197_ performed worse than 51-CRM_197_. Flow cytometry confirmed that the antibodies could bind tumor cells expressing Tn and that the antibodies from modified Tn vaccines bound more strongly than antibodies from 51-CRM_197_. Antisera were evaluated on their ability to affect CDC-mediated tumor cell killing. The results showed 74% lysis for pooled sera after 52-CRM_197_ vaccination compared to 57% after 51-CRM_197_ vaccination. Collectively, these experiments validated that fluorination, in particular the introduction of the monofluoromethyl amide, could increase the immunogenicity of the TF antigen, producing antibodies with good cross-reactivity for the native Tn antigen. This indicates a potentially productive avenue for anti-cancer vaccine development.

#### 4.2.5. Mucin-associated Thomsen–Friedenreich (TF) antigen

Mucin 1 (MUC1) is a transmembrane glycoprotein, primarily studied in mammals, including humans, mice, rats, and other species.^205^ It is presented on the surface of a multitude of cells: the respiratory tract, gastrointestinal tract, genitourinary system, *etc.* The primary function of this glycoprotein lies in providing a barrier against pathogens and other environmental factors to protect epithelial surfaces.

**Fig. 17:**
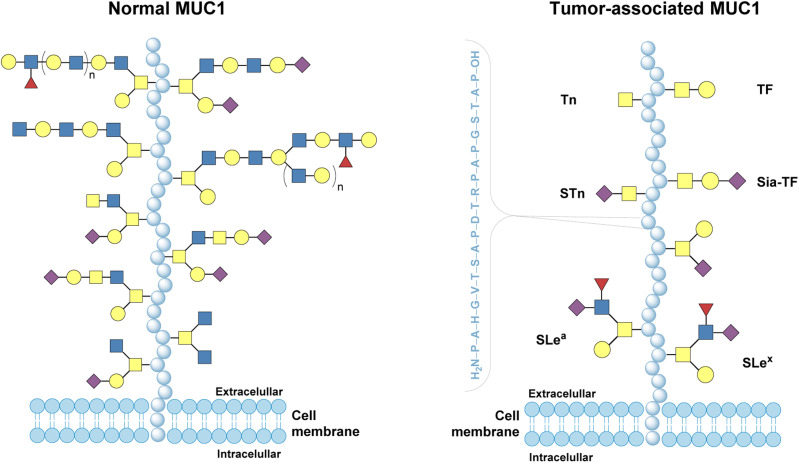
MUC1 covering normal cells (covered with complex and long carbohydrate chains masking the protein core) and tumor-associated MUC1 (aberrant glycosylation characterized by shorter and simpler carbohydrate chains).^207^

MUC1 is well documented to be overexpressed on many types of cancer cells, such as breast, pancreatic, ovarian, or lung.^179,206^ The cancer-associated glycoprotein is distinct from its healthy counterpart not only in the abundance, but also in its structural and functional characteristics (Fig. 17).^207^ MUC1 covering normal cells is heavily glycosylated: complex and long carbohydrate chains mask the protein core. In an interesting reversal of circumstances, and as a result of altered glycosyltransferase activity, on tumor tissues it undergoes aberrant glycosylation, characterized by shorter, simpler carbohydrate chains (*e.g.*, truncated *O*-glycans like T, Tn, and STn antigens). These patterns are mainly related to the extracellular region of the glycoprotein consisting of a variable number of tandem repeats, each built of 20 amino acids (the sequence PDTRPAPGSTAPPAHGVTSA). These changes expose peptide epitopes that are normally hidden, making cancer-associated MUC1 immunologically distinct. These key characteristics of MUC1 have shed light on this glycoprotein as a potential target for cancer immunological therapy.

**Fig. 18:**
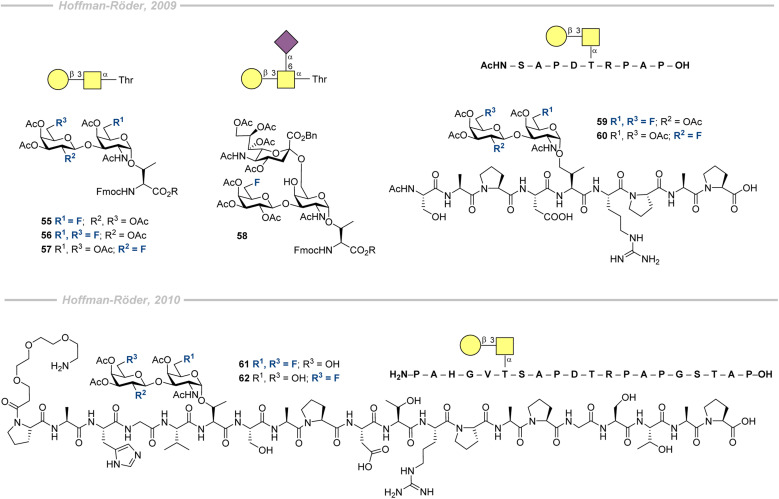
First fluorinated glycoprotein possessing a fragment of MUC1 peptide chain synthesized by Hoffmann-Röder and co-workers (top);^208^ glycoprotein mimetic including full the MUC1 repeat sequence prepared by Hoffmann-Röder and co-workers (bottom).^209^

In 2009, Hoffmann-Röder and co-authors described a route to synthesize MUC1 fragments bearing fluorinated analogues of T (55–57) and sialyl-T (58) antigens (Fig. 18, top).^208^ Di- and trisaccharides were prepared according to standard glycosylation procedures. Galactosamine building blocks were conjugated to threonine to be subsequently incorporated into a peptide chain. Compounds 56 and 57 were then submitted to automated solid-phase peptide synthesis (SPPS) according to Fmoc-strategy to be integrated into a MUC1 tandem repeat-peptide sequence comprising the immunodominant PDTRP epitope (a fragment of the peptide presented on Fig. 18). Ultimately, glycopeptides 59 and 60 were obtained following global deprotection with overall yields of 41% and 18% (based on the loaded resin), respectively.

In 2010, the group expanded their synthesis of glycopeptide analogues to create a mimetic that included the full MUC1 tandem repeat sequence (Fig. 18, bottom).^209^ Initially, fluorine-modified antigens were prepared according to standard synthetic strategies. The last step of each synthesis was deprotection of the ester of the Thr moiety for subsequent SPPS conducted according to the standard SPPS method. After the Fmoc-deprotection of the final amino acid, the triethylene glycol spacer was conjugated to the obtained sequence. Following cleavage from the resin led to the formation of 61 and 62 in 29 and 21% yields, correspondingly. Global deprotection of the glycopeptides was conducted to give the mimics in 55 and 60% yields, thereby leading to the synthesis of a convenient platform for ensuing vaccine candidates' preparation.

**Fig. 19:**
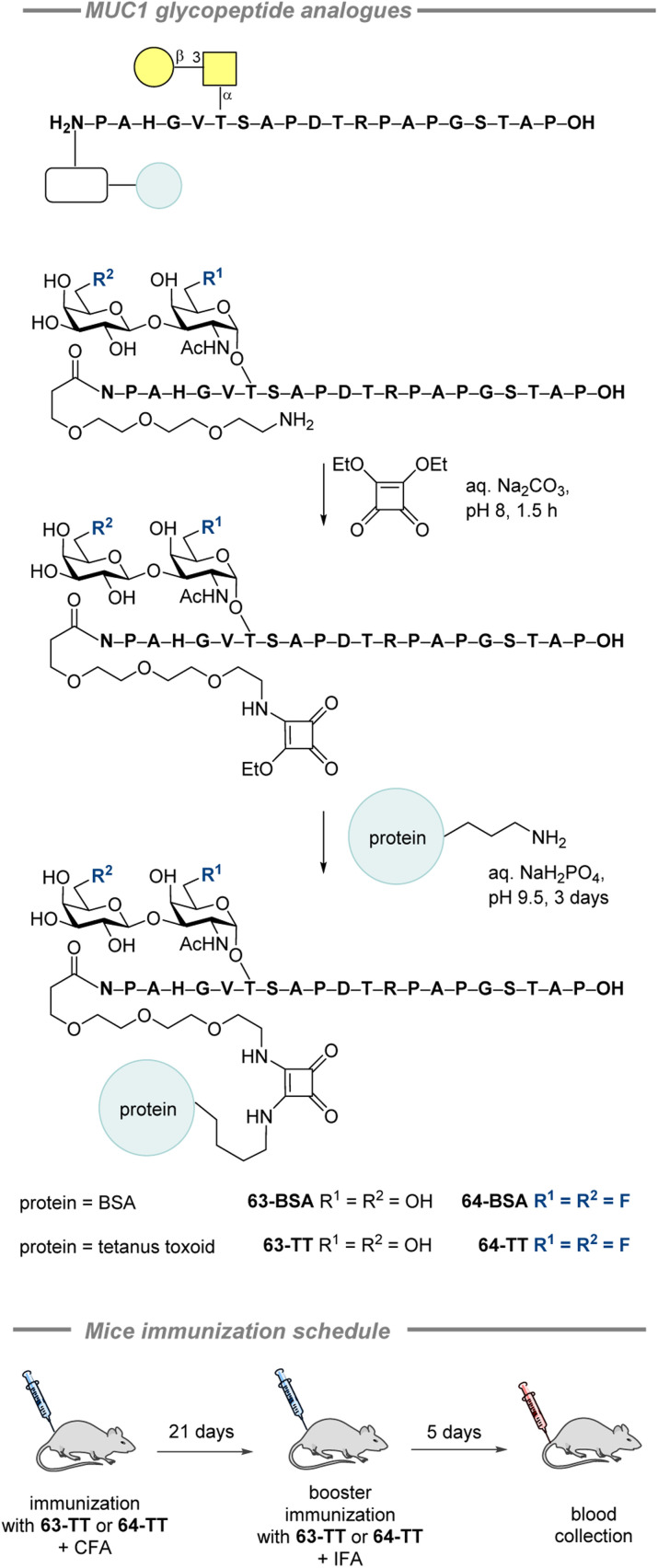
Conjugation of fluorinated and non-fluorinated MUC1 glycopeptide analogues to the carrier proteins by Kunz, Hoffmann-Röder and co-workers and the immunization schedule.^202^

In 2010, Kunz, Hoffmann-Röder and co-workers prepared a series of fluorinated and non-fluorinated MUC1 glycopeptide analogues conjugated to tetanus toxoid as vaccine candidates (64) to test if enzymatically stable fluorinated mimics could replace their less stable natural counterparts 63 (Fig. 19, top).^202^ The diethyl ester of squaric acid was used to conjugate the SPPS-synthesized glycopeptides to the carrier proteins (BSA and TT). The immunological properties of the vaccine candidate leads were evaluated *via* immunization of three balb/cJ mice with 63-TT and three mice with 64-TT. The immunization schedule is summarized in Fig. 19 (bottom). Both vaccine candidates induced high antibody levels, although the values for the fluorinated conjugate were lower. Further binding studies revealed that the antibodies formed by 63-TT strongly recognized 63-BSA, mirroring its glycopeptide sequence. Similar affinity was observed for the BSA conjugate containing 2,6-sialyl-T antigen. The antibodies induced by 64-TT showed very similar level of recognition of 63-BSA, 2,6-sialyl-T antigen, sialyl-T_N_ antigen, and the conjugate with non-glycosylated MUC1 peptide, whereas shortened to 12 amino acids the glycopeptide sequence was only recognized weakly. An important finding showing cross-reactivity potential is the equally strong binding observed for both the native conjugate 63-BSA and the fluorinated version 64-BSA. For both vaccine candidates, minimal IgM, IgA, and IgD antibody production was observed, indicating selective immune responses and initiation of immunological memory. IgG_1_ was predominant for the natural and the fluorinated candidates, whereas IgG_2a_ and IgG_2b_ levels were slightly higher for 64-TT. Moreover, the authors showed that the antibodies induced by both vaccines strongly bound to MCF-7 (human breast cancer cell line). Noteworthy, the T-antigen-MUC1 glycopeptide, as well as the corresponding difluorinated antigen, neutralized binding of the antibodies from 64-BSA and 64-TT when added to the MCF-7 cells. In contrast, MUC4, possessing a different peptide sequence, did not show any signs of neutralization, which suggests selective recognition of tumor-associated antigens.

**Fig. 20:**
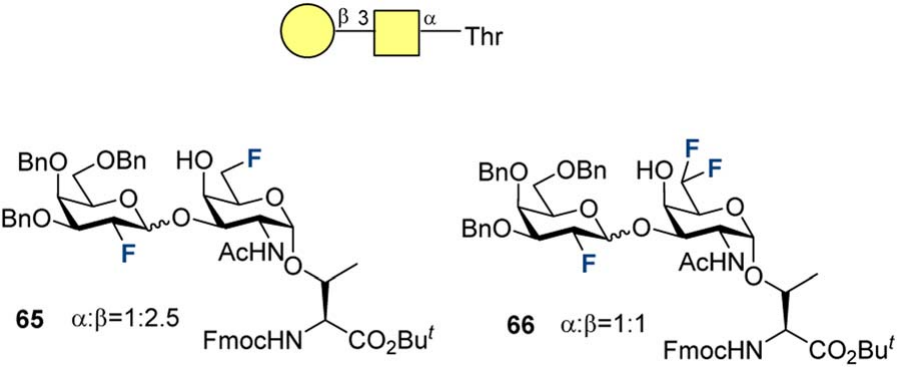
Fluorinated TF antigens by Hoffmann-Röder and co-workers.^210^

In 2011, Hoffmann-Röder and co-authors published the syntheses of two new fluorinated TF antigens (Fig. 20).^210^ The synthetic approach enabled α/β mixtures of 65 and 66 to be obtained, which were separated to isolate the β-isomers. Only antigen 65 was used in the subsequent research.

**Fig. 21:**
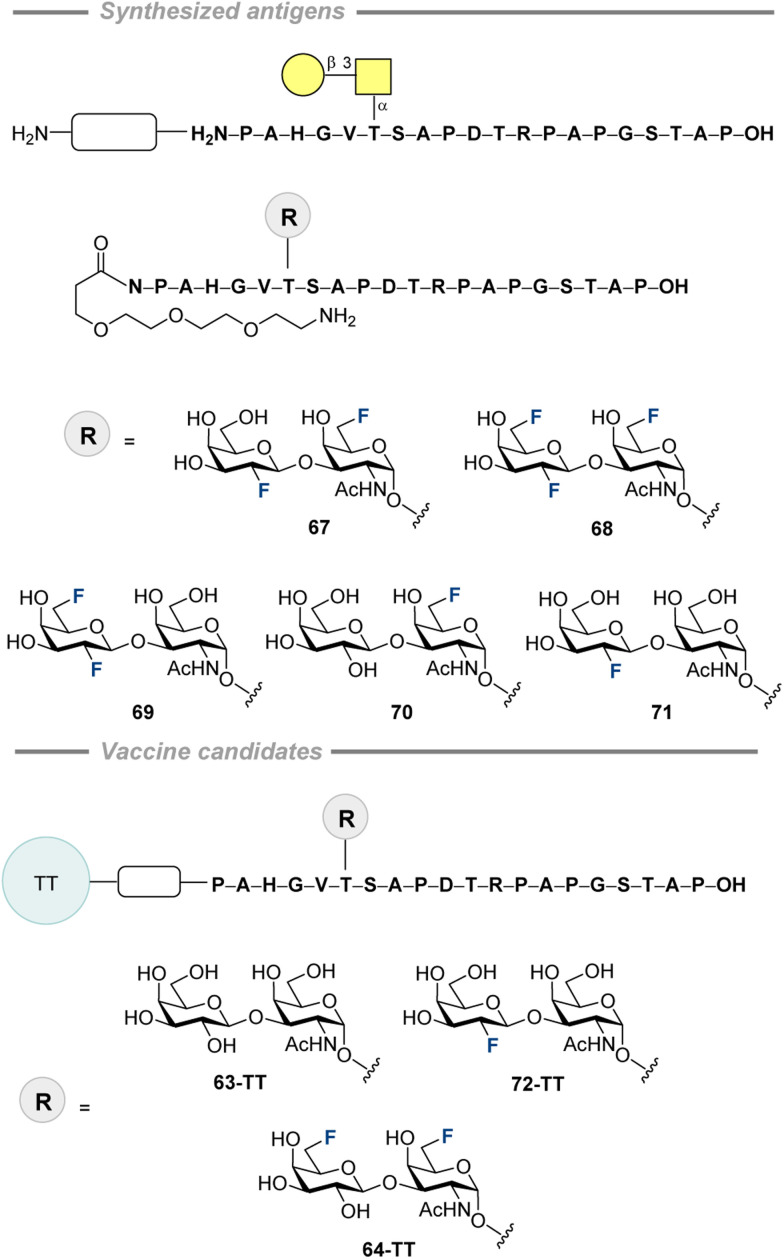
Fluorinated MUC1 antigens by Hoffmann-Röder and co-workers (top), synthetic vaccine candidates used for immunization (bottom).^211^

Later, Hoffmann-Röder and co-authors continued their investigation on TF-fluorinated antigen 67–71 development and investigated specific binding to serum antibodies induced by the vaccine candidates 63-TT, 64-TT, and 72-TT presented in Fig. 21.^211^ After preparing the fluorinated disaccharides glycosylated to Thr, standard SPPS was applied to obtain the corresponding glycoconjugates 67–71 in 15–44% yields. Neutralization experiments were performed to test if the number and position of the fluorine atoms on the glycan moieties of the glycoconjugates affect binding. For this, antibodies induced by the natural (63-TT), mono- (72-TT), and difluorinated (64-TT) glycopeptide-based vaccine candidates were applied. ELISA plates were coated with the glycopeptide-BSA conjugates reflecting the structure of the vaccines. For 63-TT, binding of all freshly prepared antibodies was similar and strong as for the native one. Monofluorinated candidate 72-TT, glycopeptides 67–69 and 71 demonstrated similar affinities, while structure 70 (no fluorine at C2′) displayed slightly lower binding. The antibodies induced by 64-TT showed similar binding with 67, 68, and 70, but 69 and 71 (no fluorine at C6) bound weaker. These recognition patterns suggest that the affinity can be affected by the fluorination pattern difference between the vaccine candidate applied to induce antibodies and the antigens. The next question to be addressed was the effect of an altered peptide sequence on the binding. To investigate this, the MUC4 glycopeptide, possessing a different peptide sequence, was tested with the antibodies induced by vaccine candidate 72-TT; no recognition was observed. These findings indicate that vaccine-induced antibodies primarily bind the whole MUC1 glycopeptide antigen, rather than its substructures like the (fluorinated) carbohydrate moiety.

**Fig. 22:**
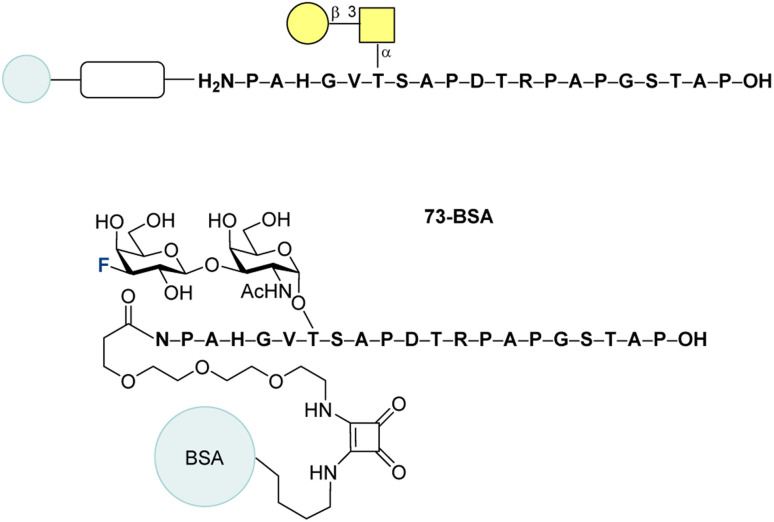
Fluorinated MUC1 antigen-BSA conjugate by Hoffmann-Röder and co-workers.^212^

In 2011, the same group described a promising new vaccine candidate 73-BSA (Fig. 22).^212^ The disaccharide 73 was synthesized as previously described and subjected to SPPS, giving the product in 50% yield. To conjugate this structure to BSA, a previously established strategy *via* diethyl squarate was employed. Next, the 73-BSA binding to the antibodies induced by the vaccine candidates 63-TT and 64-TT was investigated. The binding of the serum antibodies raised by immunization with 63-TT was partially neutralized by the natural antigen and fluorinated 73-BSA. In the case of the vaccine 64-TT, a notable difference in binding was observed. The non-fluorinated natural antigen showed the lowest affinity, with affinity increasing for the 6,6′-difluorinated antigen and reaching the highest value 73-BSA. Despite the differences in binding, the observed cross-reactivity underscores the potential of fluorinated glycopeptide conjugate 73-BSA to become a vaccine candidate.

**Fig. 23:**
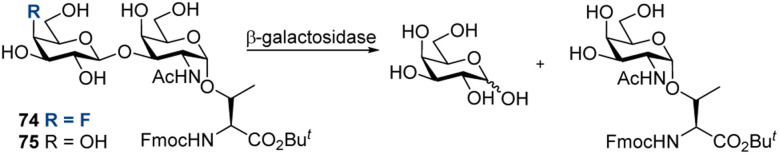
Fluorinated and non-fluorinated glycan stability test against enzymatic degradation.^203^

In 2015, Hoffmann-Röder and co-authors prepared a vaccine candidate, bearing fluorine atom at C4′ position.^203^ The synthesized fluoro disaccharide 74 and the native antigen 75 were submitted to an *in vitro* stability test against enzymatic degradation (Fig. 23). After incubation with the enzyme in aqueous 2-(*N*-morpholine)ethanesulfonic acid (MES buffer) at pH 4.5 (enzyme activity optimum) in the presence of 2,6-di-*O*-methyl-ß-cyclodextrin at 25 °C, the native antigen was half-digested in ∼3 h, while the fluorinated 74 stayed unreactive over 7 h. This clearly demonstrates the enhanced metabolic stability of the fluorinated glycan.

**Fig. 24:**
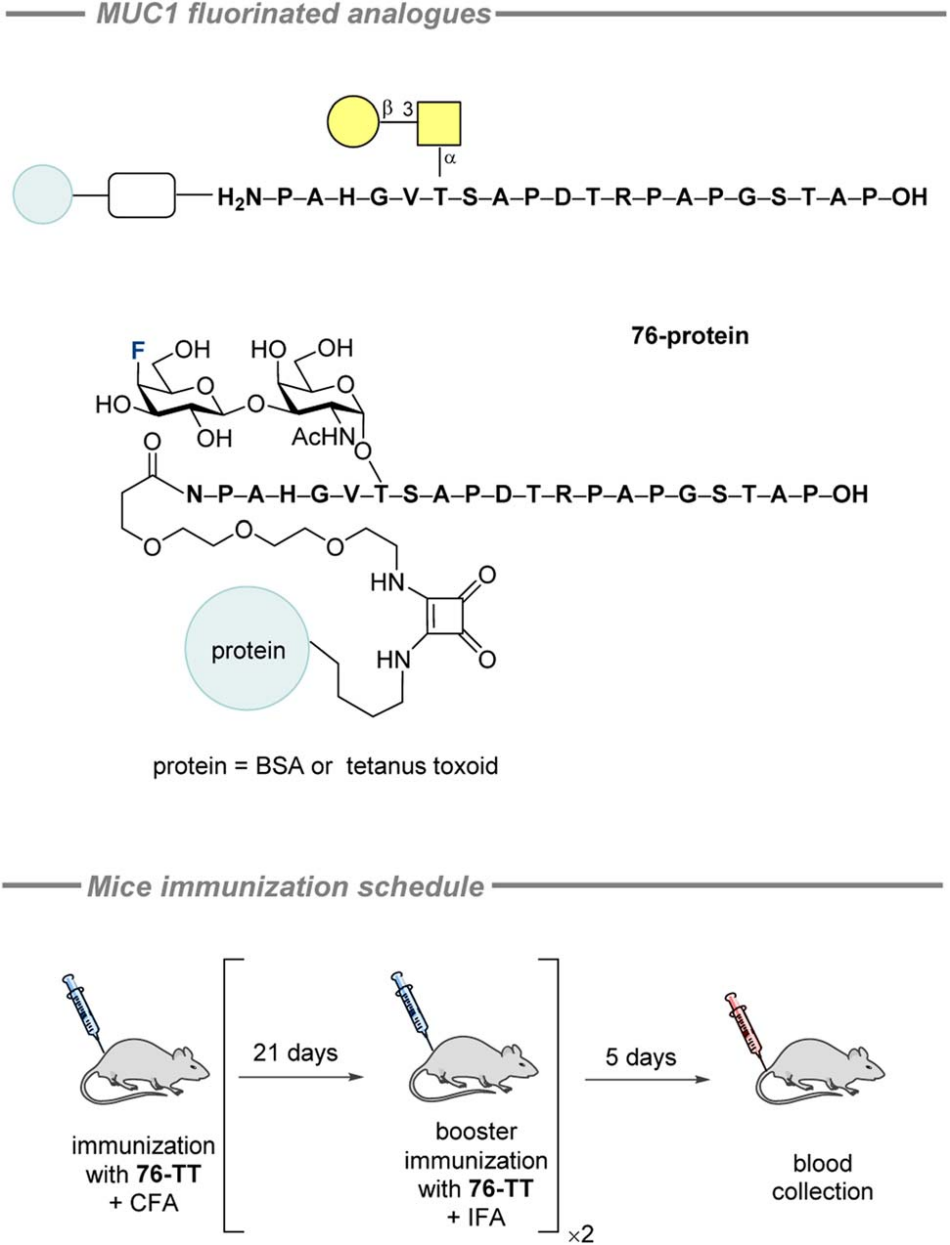
Fluorinated MUC1-protein conjugates by Hoffmann-Röder and co-workers and the mice immunization schedule.^203^

Subsequently, standard SPPS was used to synthesize the fluorinated glycopeptide 76 (13%), which was conjugated to a carrier protein *via* diethyl squarate (Fig. 24, top). The immunogenicity of the synthesized vaccine candidate was assessed by immunizing three balb/cJ mice with compound 76-TT, following the schedule detailed in Fig. 24 (bottom). Five days after the final vaccination, blood was collected, and serum antibody levels were measured using ELISA. The ELISA plates were coated with the corresponding conjugate 76-BSA. The titer level was found to be similar to the results obtained for the difluorinated vaccine 64-TT, which was used as a positive control in the current study. Carrier protein – tetanus toxoid – was tested in terms of immunogenicity as well and demonstrated a strong immune response. Isotype analysis of the antisera revealed intense formation of IgG_1_ and lower levels of IgG_2a_ and IgG_2b_. On the other hand, IgM, IgA, and IgD were produced in negligible amounts. MCF-7 binding experiments gave positive results, but it is worth to mention that the binding of antibodies from all three mice significantly differed. Additionally, the antibody binding levels measured in the sera following the first booster immunization were higher than those observed after the second booster.

#### 4.2.6. GM3

**Fig. 25:**
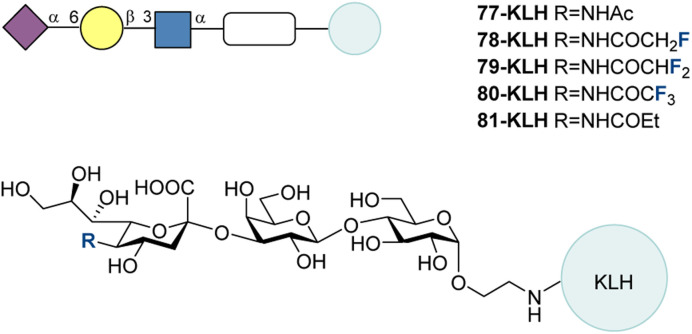
Fluorinated GM3 derivatives with various *N*-acyl modifications by Ye and co-workers.^215^

GM3 77 is one of the most frequently encountered gangliosides in human cells, and it is found on the surface of nearly all vertebrate cells.^213^ It can be overexpressed and more exposed on tumor cells, making it a prominent target for vaccine development.^214^ In 2015, Ye and co-workers synthesized a panel of GM3 derivatives with various *N*-acyl modifications to the sialic acid moiety, among which were the fluorinated motifs: monofluoromethyl (78), difluoromethyl (79), and trifluoromethyl (80) amides (Fig. 25). Among the non-fluorinated analogues, the ethyl amide 81 performed particularly well in immunological evaluation.^215^ These glycans were conjugated to KLH and used to vaccinate mice, without an adjuvant.

After 4 vaccinations in accordance with the standard scheme (Fig. 12, bottom), the anti-GM3 IgG titers were quantified. The non-fluorinated 81-KLH led to the highest titer, 3.7 times greater than that of 77-KLH. The second highest IgG titer resulted from 79-KLH, 1.6 times greater than for 77-KLH. These results showed that the difluoromethyl compound 79 could be investigated as a potential vaccine, however the ethyl compound 81 induced a stronger IgG response in this investigation. This did illustrate that certain modifications led to increased immunogenicity compared to the native structure 77 and that some of these structures afforded antibodies with a cross reactivity to 77 that could indicate promising vaccine candidates.

#### 4.2.7. Globo H

Globo H (82) is a key TACA with significant implications in cancer diagnosis and immunotherapy. It is overexpressed in a wide range of cancers, including: breast, prostate, ovarian, colorectal, and lung cancer.^216^ Synthetic Globo H based vaccines have progressed to various stages of clinical studies.^217^ Despite the promising start, early-stage clinical results demonstrated that the level of IgG antibodies induced by the vaccine candidates was significantly lower than IgM antibodies.

**Fig. 26:**
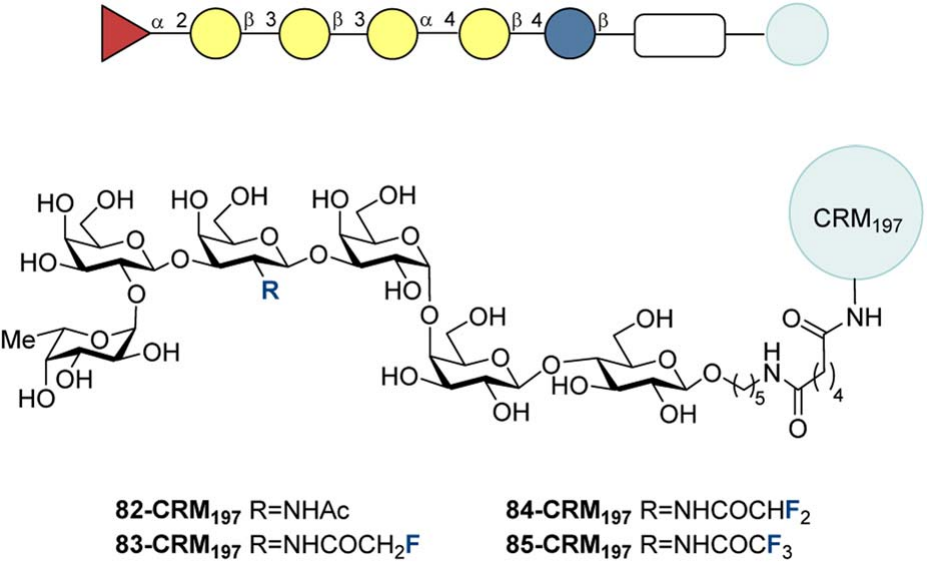
Fluorinated Globo H-CRM_197_ conjugates with *N*-acyl modifications by Ye and co-workers.^218^

In 2021, Ye and co-workers replaced the methyl moiety of the native acetamide of 82 with a monofluoromethyl group (83), difluoromethyl group (84) and a trifluoromethyl group (85) (Fig. 26).^218^ Conjugates with CRM_197_ were used as vaccines for mice with C34 as the adjuvant following the standard scheme (Fig. 12, bottom). 84-CRM_197_ and 85-CRM_197_ led to significantly higher anti-Globo-H IgG titers compared to 83-CRM_197_. Antisera from 84-CRM_197_ and 85-CRM_197_ vaccinated mice led greater cell lysis towards MCF-7 cancer cells by complement-dependent cytotoxicity (CDC).

**Fig. 27:**
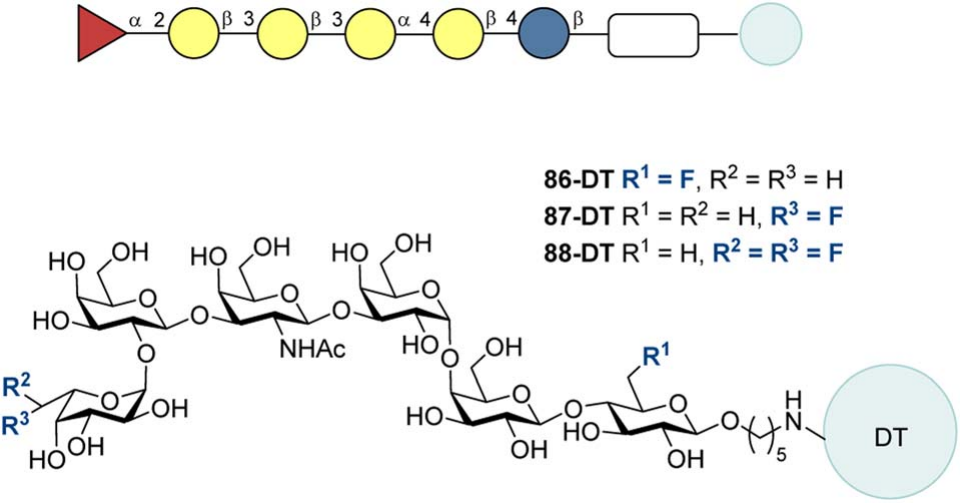
Globo H fluorinated analogues by Wong and co-workers.^217^

Wong and co-authors have demonstrated that altering the carrier and the adjuvant used can induce a strong immune response with the antibody class switching from IgM to IgG in case of Globo H and Globo H-related epitopes.^219^ In light of these promising findings, the group decided to investigate the impact of structure modifications with different substituents. Fluorine was incorporated at the C-6 position of Glc at the reducing end (86) or the C-6 position of Fuc at the nonreducing end of Globo H (87) (Fig. 27).^217^ Corresponding hexasaccharides bearing an amine-linker at the reducing end were prepared through chemoenzymatic synthesis strategies. Unfortunately, an attempt to obtain difluoro-substituted molecule 88 failed giving only trace amounts of the desired product. Molecules 86 and 87 were conjugated to the DT (carrier protein) *via p*-nitrophenyl adipate diester and later combined C34 adjuvant. For immunological investigation 10 groups of 5 female balb/c mice were immunized intramuscularly with Globo H analogues 86-DT and 87-DT according to the Fig. 12 (bottom). The collected sera were tested on the glycan microarray consisting of 94 different TACAs.

Antibodies generated by the tested candidates were recognized by Globo H, Globo H analogues, and Globo H fragments, but not by other TACAs and functional linkers. As Globo H, Gb5, and SSEA4 were found to be overexpressed on breast cancer stem cells and possess rather similar core structures, these TACAs were selected as standard antigens for the DT-conjugates. In the binding tests of antibodies induced against the chosen antigens high IgG antibodies titer, indicating a T-cell-dependent immune response, was obtained. No significant IgM antibody formation was indicated in case of both candidates. IgG tests revealed regioselectivity of the immunogenicity modulation by the fluorine substituents. The F moiety at the C-6 position of Glc at the reducing end of Globo H (86-DT) demonstrated a comparable titer to the natural Globo H, whereas 87-DT, bearing F group at the C-6 position of Fuc at the nonreducing end of Globo H, showed significantly weaker interaction with Globo H. Rather similar results were obtained during the studies with Gb5 and SSEA4. The ratio of IgG/IgM for 86-DT and 87-DT was found to be 78.07 and 11.28, respectively. IgG subclass test showed that the antibodies had a significant amount of IgG_1_, IgG_2b_, IgG_2c_, and IgG_3_ and low level of IgG_2a_. It is noteworthy that the glycans bearing the azide-group instead of the fluorine-atom performed better.

#### 4.2.8. KH-1

**Fig. 28:**
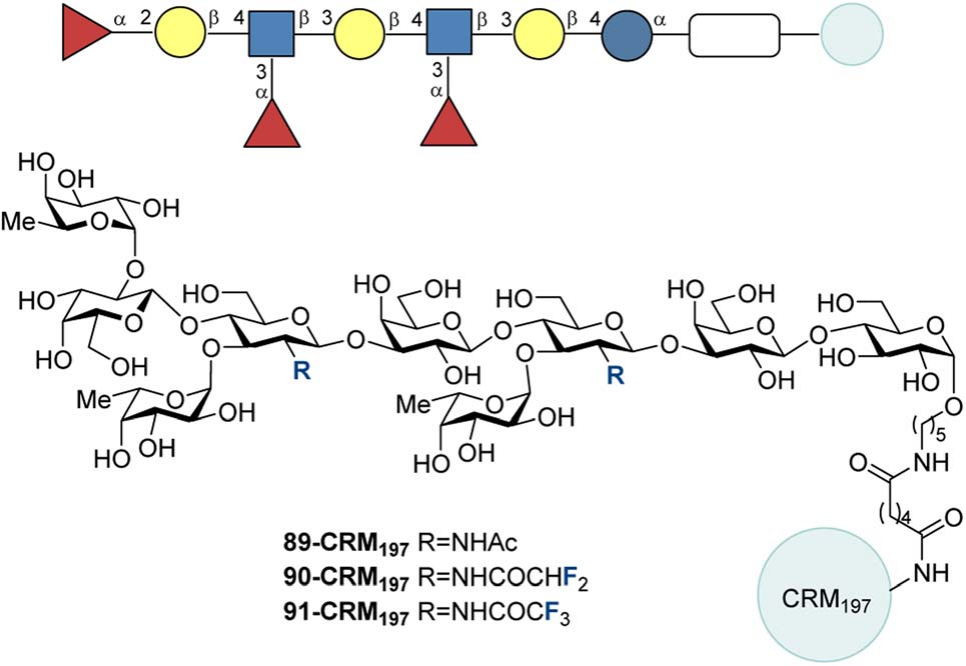
CRM_197_ conjugated fluorinated analogues of KH-1 by Ye and co-workers.^220^

In 2023, Ye and co-workers synthesized CRM_197_ conjugated analogues of KH-1 where the methyl moiety of the native acetamide of 89 was replaced by a difluoromethyl group (90) and a trifluoromethyl group (91) (Fig. 28).^220^ Mice were vaccinated 4 times with glycoconjugates (Fig. 12, bottom) and Freund's adjuvant then anti-KH-1 IgG titers were determined. The titer for 91-CRM197 was 26 times greater than that of 89-CRM197. 90-CMR197 led to 2.2 times higher anti-KH-1 IgG titers than the native structure. Analysis of the IgG subtypes revealed a mixed Th1/Th2 response by the presence of IgG_1_, IgG_2a_, IgG_2b_, and IgG_3_. The IgG antibodies were capable of binding native KH-1 on MCF-7 cancer cells, with the antibodies from the fluorinated analogues displaying a higher binding affinity than those from 89-CRM_197_.

#### 4.2.9. Pentamannose

**Fig. 29:**
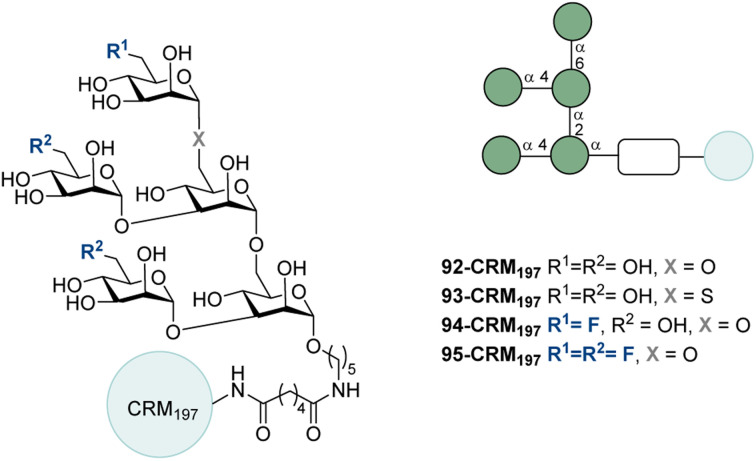
Natural Man_5_, an S-linked analogue and fluorinated analogues by Ye and co-workers.^223^

The Man_5_ motif is found on the gp120 protein of HIV and it therefore logically serves as an appealing target for vaccine lead validation.^221^ Previous carbohydrate vaccine investigations focused on Man_9_ without success.^222^ In 2022, Ye and co-workers synthesized the natural Man_5_ (92), an S-linked analogue (93), a monofluorinated analogue (94), and a trifluorinated analogue (95) (Fig. 29).^223^ These modifications were with the aim of increasing immunogenicity whilst enabling cross reactivity of the antibodies with native Man_5_. The glycans were conjugated to CRM_197_ and used to vaccinate mice with ODN1826 as an adjuvant (Fig. 12, bottom). 92-CRM_197_ did not lead to production of Man_5_-specific antibodies. The results of 93-CRM_197_, 94-CRM_197_ and 95-CRM_197_ were identical in that just 1 of the 6 mice produced significant amounts of anti-Man_5_ antibodies. The antisera bound 92-CRM_197_ strongly, indicating that the antibodies were directed mainly towards CRM_197_.

#### 4.2.10. Summary

Fluorinated carbohydrate-based therapeutic cancer vaccines are part of an emerging field in immunological studies. A wide range of TACAs have been synthetically modified: Tn, STn, TF, sialyl-TF, MUC1, GM3, Globo H, KH-1, and a pentamannose epitope. The amide moiety of these molecules has been systematically modified with fluoroacetyl groups with different fluorination patterns by Ye and co-workers and this has now matured into a valuable design strategy. Studies have revealed that the effect of modification is highly target-dependable. The concept has been demonstrated in the MUC1 glycopeptide by Hoffmann-Röder and colleagues. Reproducible synthetic approaches to modified MUC1 have been validated and this lowers the barrier to immunological investigations. Glycopeptides with varying sites of fluorination have been tested in the context of both antibody formation and recognition. Comparatively, fluorine incorporation into carbohydrate antigens affected immunogenicity much more than in the case of glycopeptide (MUC1). This may be a consequence of the less pronounced changes caused by fluorine atoms in the interior of the glycopeptide. Wong and co-workers’ work on Globo H analogues serves to demonstrates the importance of the modification site choice in the initial design of the vaccine. Overall, strategic incorporation of fluorine atoms into antigen structures continues to enrich the goal of immunogenicity modulation and induction of potent cross-reactive antibodies in certain cases.

## 5. Conclusions and future directions

Carbohydrate-based vaccines continue to play a foundational role in public health strategies across the globe. Pathogens that are familiar to us all include *Streptococcus pneumoniae*, *Haemophilus influenzae*, and *Neisseria meningitidis*, which are omnipresent and continually trying to evade detection by our immune system. There is a risk of taking vaccines for granted and it is hoped that this short Perspective serves to highlight the challenges and opportunities that exist in this important area of contemporary research. Carbohydrates are a crucially important structural sub-set of vaccine design modules, and their clinical success is grounded in the diversity and complexity that they afford. The natural role of glycans in modulating molecular and cellular recognition events renders these biomolecules ideally suited to the task of triggering the immune system, and moving to unnatural analogues offers the opportunity to further enhance immunogenicity and fine-tune their physicochemical profiles. Broadening the spectrum of available antigens may reveal new correlations and causations, thereby expediting rational design in the future. Although the validation of a safe and fully functional vaccine is incredibly complex, looking at epitope design through the lens of fluorination offers a new and exciting perspective. What is clear is that the introduction of fluorine into carbohydrate-based vaccine leads can lead to discernible patterns that enable structure–activity relationships to be extrapolated.

## Author contributions

The manuscript was written with contributions from all authors.

## Conflicts of interest

There are no conflicts to declare.

## List of abbreviations

CPS: Capsular polysaccharide
TACA: Tumor-associated carbohydrate antigen
APC: Antigen-presenting cell
DC: Dendritic cell
MHC: Major histocompatibility complex
Th: T helper
BCR: B cell receptor
CRM_197_: Diphtheria toxin mutant
TT: Tetanus toxoid
DT: Diphtheria toxoid
OMPC: Meningococcal outer membrane protein complex
rEPA: *Pseudomonas aeruginosa* exotoxin A
BSA: Bovine serum albumin
KLH: Keyhole limpet hemocyanin
Hib: *Haemophilus influenzae* type b
AGA: Automated glycan assembly
LOS: Lipooligosaccharide
PorA: Porin A
Alum: Aluminium hydroxide adjuvant
ST: Serotype
ELISA: Enzyme-linked immunosorbent assay
CDC: Complement dependent cytotoxicity
ADCC: Antibody dependent cellular cytotoxicity
MUC1: Mucin 1
CFA: Complete Freund's Adjuvant
IFA: Incomplete Freund's Adjuvant
ODN: Oligonucleotide
Tn: Thomsen-nouveau
STn: Sialyl-Thomsen-nouveau
TF: Thomsen–Friedenreich
sialyl-TF: Sialyl-Thomsen–Friedenreich
PSA: Polysialic acid

## Data Availability

The data underlying this study are available in the published article.
